# Recent Progress in Optical Frequency-Domain Reflectometry: Performance Enhancement, Emerging Applications and Machine Learning Integration

**DOI:** 10.3390/s26144397

**Published:** 2026-07-10

**Authors:** Zhancong Xu, Yufeng Wang, Qirui Huang, Feiyu Jiao, Yansong Du, Yuting Zhou, Bangyao Wang, Longfei Yu, Faisal Nadeem Khan, Xun Guan

**Affiliations:** Shenzhen International Graduate School, Tsinghua University, Shenzhen 518055, China

**Keywords:** optical frequency-domain reflectometry (OFDR), distributed optical fiber sensing (DOFS), battery health monitoring, machine learning

## Abstract

Optical frequency-domain reflectometry (OFDR) has become a core technology in distributed optical fiber sensing (DOFS) based on its ultra-high spatial resolution down to the sub-millimeter scale and excellent measurement sensitivity. However, its practical performance is significantly limited by various non-ideal factors, and existing reviews still lack systematic discussion on its latest research directions. This paper first introduces the fundamental principles and core error mechanisms of OFDR then summarizes key technological advances in its performance enhancement over the past five years. We further review its multi-scenario applications with a special focus on battery health monitoring and analyze the integration of machine learning in OFDR signal processing. Finally, we discuss the key challenges and future research prospects in this field.

## 1. Introduction

Distributed optical fiber sensing (DOFS) technology enables continuous, fully distributed spatial sampling along a single optical fiber. With inherent advantages including long-distance monitoring capability and electromagnetic interference immunity, it has become a core sensing solution for critical fields such as infrastructure structural health monitoring (SHM), energy system safe operation and advanced equipment condition monitoring. In recent years, the growing demand for high-density, high-precision and multi-physics coupled monitoring has exposed limitations of traditional point-type or quasi-distributed sensing in terms of spatial continuity and coverage efficiency. This trend has driven the continuous evolution of DOFS technologies based on Rayleigh, Brillouin and Raman scattering effects. Among these technologies, optical frequency-domain reflectometry (OFDR) stands out with its ultra-high spatial resolution down to the sub-millimeter scale and excellent measurement sensitivity. It is evolving from a conventional diagnostic tool for fiber links into a high-precision distributed sensing platform adaptable to complex engineering scenarios.

The core measurement principle of OFDR was first proposed and experimentally verified by Eickhoff and Ulrich in 1981, laying the basis for its subsequent systematic development [1]. At that time, optical time-domain reflectometry (OTDR) was the dominant method for fiber link characteristic evaluation. OTDR achieves spatial localization by transmitting optical pulses and detecting the arrival time of backscattered echoes. However, its spatial resolution is fundamentally limited by pulse width and system signal-to-noise ratio (SNR), making it unable to achieve a balanced performance between high spatial resolution and measurement accuracy. In contrast, OFDR acquires the fiber backscattering distribution via coherent detection based on a frequency-swept light source. It readily achieves ultra-high spatial resolution in short- to medium-range measurement scenarios while providing abundant phase and spectral information. This makes it particularly suitable for precise detection of local micro-structural changes, micro-strain and micro-temperature variations.

With advances in tunable laser sources (TLSs), data acquisition and signal processing, OFDR has gradually diversified into several application-specific implementation forms. For instance, Rayleigh backscattering (RBS) spectral fingerprint-based OFDR enables distributed temperature and strain measurement by tracking local RBS spectral shifts. Phase-sensitive OFDR (φ-OFDR) further exploits phase information for high-sensitivity demodulation of fast perturbations such as acoustic waves and vibrations. To enhance engineering deployability, polarization and vector detection schemes and time-gated digital OFDR have been developed to mitigate fading, enhance multi-parameter decoupling and extend sensing range. All these technical routes aim to optimize performance trade-off among spatial resolution, measurement range, dynamic range, real-time performance and deployability.

However, the high-accuracy performance of OFDR systems remains significantly limited by various non-ideal characteristics. Key error sources include TLS frequency sweep non-linearity, laser phase noise and system chromatic dispersion. These errors may degrade the spatial point spread function (PSF), reduce SNR and cause accumulation of demodulation deviation, collectively restricting OFDR’s performance ceiling. To address these challenges, relevant technologies targeting key issues including non-linearity calibration, noise suppression, demodulation algorithm optimization and real-time implementation have advanced rapidly and received extensive attention in the past five years.

Meanwhile, two notable emerging trends in OFDR applications deserve particular focus. First, machine learning (ML) methods are increasingly integrated into OFDR signal processing. Learning models have been used for noise suppression and other tasks, sometimes with incorporated physical priors to improve cross-scenario generalization, aiming to extract high-value features inaccessible to conventional methods and break through performance limits. Second, the urgent demand for safety improvement and lifetime assessment in new energy and energy-storage systems has made battery health monitoring an important emerging DOFS application. Batteries exhibit strong thermo-mechanical coupling during charge-discharge cycles and aging, with hot spot generation and abnormal strain distribution directly linked to safety risks and performance degradation. OFDR’s ultra-high spatial resolution and embeddable sensing capability make it uniquely suited for in situ distributed temperature and strain monitoring in batteries, supporting integrated battery health management frameworks combining sensing, diagnosis and prognosis.

Numerous reviews on OFDR technology and applications have been published in recent years. Qu et al. [2] reviewed OFDR’s latest progress and core performance improvement paths from a distributed sensing perspective. Ding et al. [3] summarized OFDR-based DOFS application scenarios and discussed photonic integrated chip development trends. Lobach et al. [4] outlined OFDR performance enhancement strategies from the standpoint of error mechanisms and signal processing, noting potential fusion directions with OTDR. Reyes-Vera et al. [5] proposed a research agenda for ML in fiber sensing, providing reference for reproducible and generalizable data-driven method engineering implementation. Zhai et al. [6] focused on 3D shape sensing and summarized OFDR-inclusive reconstruction routes and data-driven methods’ benefits for accuracy and efficiency.

While these works have established a solid foundational framework for OFDR research, they are constrained by their publication timelines or narrow thematic focuses. In particular, the most transformative advances in OFDR-ML deep integration and operando battery health monitoring, which have emerged as core research hotspots in recent years, have not yet received systematic and unified treatment. For instance, the machine learning-focused review by Reyes-Vera et al. [5] outlines a general cross-field research agenda rather than analyzing OFDR-specific signal processing pipelines and performance breakthroughs, whereas operando battery health analytics has rarely been systematically addressed in prior comprehensive OFDR overviews. This review bridges these critical gaps by providing a timely, holistic analysis that explicitly connects core OFDR physics with these emerging, high-impact domains.

**Figure 1:**
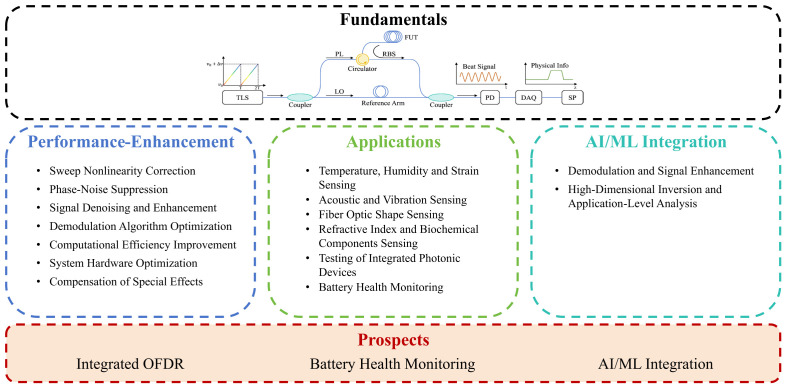
Structure diagram of this review.

Accordingly, this paper first presents a comprehensive review of the fundamental measurement principle and core error mechanisms of OFDR. We then systematically summarize the latest advances in OFDR performance improvement technologies over the past five years. Moreover, this review organizes emerging methods that integrate artificial intelligence (AI) and ML into OFDR data analysis and system optimization and discusses new application directions such as battery health monitoring and integrated OFDR. The structure diagram of this review is shown in Figure 1. The remainder of this paper is organized as follows. Section 2 introduces OFDR fundamentals and primary error sources. Section 3 summarizes key technologies and algorithms for improving OFDR performance. Section 4 reviews the multi-scenario applications of OFDR, with a special focus on battery health monitoring. Section 5 discusses the latest research progress in the integration of AI and ML in the OFDR field. Finally, Section 6 concludes the whole review and prospects the future research directions.

## 2. Fundamentals and Error Analysis of OFDR

### 2.1. Fundamental Principle of OFDR Sensing

DOFS encodes external physical perturbations into transmitted light parameters via light–fiber medium interactions and recovers spatially resolved distributed measurands through signal demodulation. The ultra-high spatial resolution of OFDR distributed sensing is fundamentally rooted in the elastic RBS effect in optical fibers. This effect originates from the elastic interaction between incident photons and microscopic refractive index inhomogeneities formed in the fiber core during fabrication. The resulting backscattered light retains the probe light frequency, is continuously distributed along the fiber and exhibits high backward coherence. These inherent properties form a unique and repeatable Rayleigh coherent spectral fingerprint for each spatial position along the fiber, which constitutes the core physical basis for OFDR’s high-precision distributed sensing.

**Figure 2:**
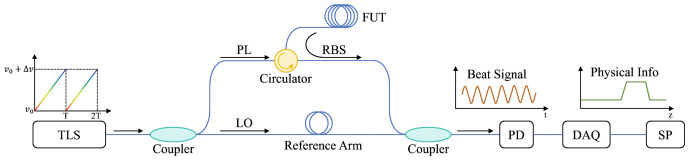
Basic OFDR system schematic. TLS: tunable laser source (the color gradient schematically illustrates the frequency tuning characteristic and does not encode quantitative frequency values), LO: local oscillator, PL: probe light, FUT: fiber under test, RBS: Rayleigh backscattering, PD: photodetector, DAQ: data acquisition, SP: signal processing.

OFDR is the optical implementation of frequency-modulated continuous-wave (FMCW) interferometric ranging. A typical OFDR system adopts a Mach–Zehnder or Michelson two-beam interferometric architecture, consisting of a high-coherence TLS, fiber couplers, a fiber under test (FUT), a fixed-length reference arm, photodetector (PD), high-speed data acquisition (DAQ) modules and a digital signal processing unit, as illustrated in Figure 2. The TLS outputs an optical field with approximately linear and continuous frequency tuning over a single sweep period, with instantaneous optical frequency expressed as:

(1) $$v\left(t\right)=v_{0}+\gamma t,$$

where $v_{0}$ is the initial optical frequency, γ is the sweep rate and v(t) denotes the instantaneous optical frequency at time *t*. The corresponding output optical field can be written as:

(2) $$E_{LO}\left(t\right)=E_{0}exp\left\{j\left[2\pi v_{0}t+\pi \gamma t^{2}+\varphi \left(t\right)\right]\right\},$$

where $E_{0}$ is the field amplitude and φ(t) represents random phase noise introduced by the laser linewidth and environmental perturbations. The TLS output is split into two beams via a fiber coupler. One beam serves as the local oscillator light (LO, i.e., reference light) transmitted directly to the PD, while the other acts as probe light launched into the FUT. The RBS signal from the FUT returns along the original path and recombines with the LO light to generate coherent interference.

Each spatial position *z* along the FUT has a unique RBS coefficient R(z), determined by local refractive index inhomogeneities. The round-trip propagation delay for light reaching position *z* is given by

(3) $$\tau =\frac{2nz}{c},$$

where *n* is the effective refractive index of the fiber and *c* is the speed of light in vacuum. The RBS optical field from position *z* can thus be written as:

(4) $$E_{s}\left(t,z\right)=\sqrt{R(z)}E_{0}exp\left\{j\left[2\pi v_{0}\left(t-\tau \right)+\pi \gamma {\left(t-\tau \right)}^{2}+\varphi \left(t-\tau \right)\right]\right\}.$$

The LO and backscattered signal light interfere coherently on the photosensitive surface of PD, which converts the interference intensity into a heterodyne beat electrical signal. The total detected light intensity can be expressed as:

(5) $$I\left(t\right)=|E_{LO}\left(t\right)+E_{s}{\left(t,z\right)|}^{2}.$$

Substituting Equations (2) and (4) into Equation (5) and expanding, the detected signal can be separated into direct current (DC) and alternating current (AC) components. After DC component removal, the AC beat signal is:

(6) $$I_{AC}\left(t\right)=2\sqrt{R(z)}cos\left[2\pi \left(f_{b}t+v_{0}\tau -\frac{1}{2}\gamma \tau^{2}\right)+\varphi \left(t\right)-\varphi \left(t-\tau \right)\right].$$

For linewidth-dominated phase diffusion, the phase difference term φ(t)−φ(t−τ) arises from the laser’s random phase noise, and its variance approximately increases with the propagation delay τ (and thus the sensing distance *z*). This cumulative effect leads to progressive coherence degradation between the local oscillator and backscattered signals at longer distances, which is the primary limiting factor for long-range high-accuracy OFDR measurements. This physical limitation directly motivates the advanced phase-noise suppression techniques reviewed in Section 3.2.

The instantaneous beat frequency $f_{b}$ is defined as the time derivative of the deterministic interference phase term divided by 2π. Substituting the round-trip delay expression $\tau =\frac{2nz}{c}$ (Equation (3)) into this definition yields the core spatial mapping relationship:

(7) $$f_{b}=\frac{1}{2\pi }\frac{d\Phi (t)}{dt}=\frac{2n\gamma z}{c}.$$

This equation shows a linear relationship between beat frequency and spatial position *z* along the FUT, which forms the basis for OFDR spatial localization. By applying fast Fourier transform (FFT), the time-domain beat signal is transformed into the frequency domain, and the beat frequency is then mapped to spatial distance to obtain the backscatter trace along the FUT.

It should be emphasized that the above mapping relies on the ideal premise that the sampled signal is uniformly spaced on the optical frequency (or wavenumber *k*) axis. In practical systems, however, TLS sweep output inevitably deviates from the ideal linear ramp, causing a non-linear relationship between instantaneous optical frequency and sampling time. Fixed-interval time-domain sampling thus leads to non-uniform sampling on the optical frequency axis, resulting in spatial localization deviation. To address this issue, auxiliary frequency reference modules such as a fixed-path auxiliary interferometer (AUX) or gas absorption cell are widely adopted for real-time instantaneous optical frequency calibration. The main interferometer signal is then resampled to achieve uniform sampling on the wavenumber axis, which is a core prerequisite for high-precision OFDR measurements.

The key performance metrics of an OFDR system mainly include spatial resolution and maximum measurable sensing range, which are inherently coupled and require performance trade-offs. Taking the differential of both sides of Equation (7) gives the quantitative relationship between spatial resolution and spectral resolution:

(8) $$\delta z=\frac{c\delta f_{b}}{2n\gamma }.$$

Here, the spectral resolution $\delta f_{b}$ is primarily limited by the sampling process. In general, the sampling duration equals the single-sweep period of the TLS. Substituting this relationship into Equation (8) gives the theoretical limit of spatial resolution determined by the laser sweep span Δv:

(9) $$\delta z=\frac{c}{2n\gamma }\cdot \frac{1}{T}=\frac{c}{2n\Delta v}.$$

Equations (8) and (9) provide the theoretical upper limit of the spatial resolution of an OFDR system, which is fundamentally governed by the optical hardware (e.g., the laser sweep span). To ensure terminological rigor, this hardware-limited resolution (δz) is defined as the physical spatial resolution. However, practical systems rarely reach this theoretical limit, as spectral shift estimation requires sliding window extraction of local backscattering signals in the distance domain followed by cross-correlation (CC) processing. For a correlation window of length *N*, the effective practical spatial resolution, which represents the actual spatial density of the demodulated metrological data (such as strain and temperature), is defined as the sensing spatial resolution (ΔZ):

(10) ΔZ=Nδz.

To maintain conciseness and align with the general convention in the distributed sensing literature, the term “spatial resolution” in the subsequent sections of this review implicitly refers to the sensing spatial resolution (ΔZ), unless the physical spatial resolution (δz) is explicitly specified.

Similarly, from Equation (7), the theoretical maximum sensing distance of an OFDR system is derived as:

(11) $$L_{max}=\frac{c}{2n\gamma }f_{b}^{max}=\frac{cf_{s}}{4n\gamma },$$

where $f_{s}$ is the sampling frequency. According to the Nyquist sampling theorem, the highest unaliased beat frequency is limited by $f_{s}/2$. Since the beat frequency increases linearly with distance, as expressed in Equation (7), the maximum measurable distance is constrained by the highest beat frequency that can be recorded and processed without aliasing, leading to Equation (11). Therefore, extending the sensing range requires either a higher acquisition bandwidth or a lower sweep rate γ. However, this range extension is intrinsically coupled with other system parameters. The physical spatial resolution is determined by the laser frequency excursion Δv, as shown in Equation (9), reflecting the Fourier-transform-limited nature of OFDR ranging. If the sweep duration *T* is kept constant, reducing γ directly reduces Δv=γT, thereby degrading the physical spatial resolution. Conversely, maintaining the same frequency excursion at a lower sweep rate requires a longer sweep duration, which reduces the update rate and increases the amount of acquired data. Consequently, long-range OFDR does not only require sufficient laser coherence but also imposes stringent requirements on acquisition bandwidth, data throughput and computational processing capability.

**Figure 3:**
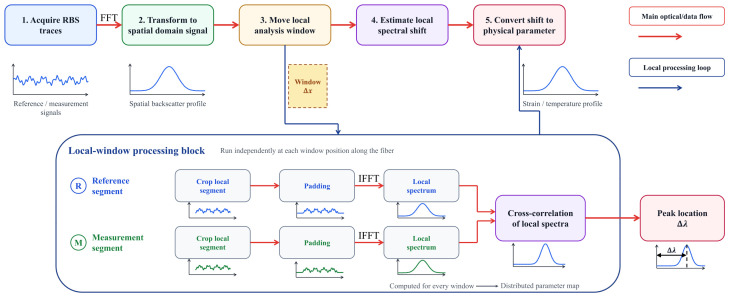
Demodulation workflow of OFDR based on RBS spectral.

When the fiber is subjected to external physical perturbations, the local RBS spectrum at the corresponding position will undergo a linear shift. Therefore, the extraction of perturbation information from local Rayleigh spectral features is the core of OFDR distributed sensing, and the demodulation algorithm directly determines system sensing performance. At present, the most widely used scheme is the classic spectral shift correlation demodulation method proposed by Froggatt et al. in 1998 [7], as illustrated in Figure 3.

In a typical demodulation workflow, the system first acquires the beat signal traces in the unperturbed reference state and perturbed measurement state, respectively. A sliding window extracts local RBS signals in the distance domain, and inverse FFT (IFFT) converts the windowed signal to the optical frequency domain to obtain the local Rayleigh spectral fingerprint. CC or spectral registration algorithms estimate the spectral shift between the two states, which is finally mapped to strain and temperature changes via pre-calibrated coefficients.

Notably, the sliding window length directly governs the trade-off between spatial resolution and demodulation robustness. A longer window provides richer spectral information for more robust shift estimate but significantly reduces the effective spatial resolution. Conversely, a shorter window improves spatial resolution but decreases spectral overlap between two states and increases noise sensitivity, leading to a higher risk of spurious peaks and misdetections. Window parameters should therefore be selected according to the measured object characteristics and target measurement accuracy.

**Figure 4:**
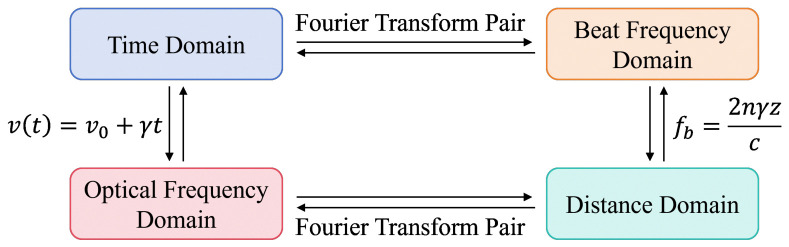
Linear mappings and Fourier-transform relations among time domain, beat-frequency domain, distance domain and optical-frequency domain.

In addition, the complete spectral demodulation process involves mutual transformations among four core domains, namely the time domain, beat frequency domain, distance domain and optical frequency (or wavenumber) domain. The linear mapping relationships and Fourier transform pairs between these domains are shown in Figure 4.

For clarity in this review, three commonly used operating/demodulation modes of OFDR can be distinguished within the common Mach–Zehnder/Michelson interferometric architecture with linear frequency tuning, each optimized for different application scenarios. A clear distinction among these modes is essential to avoid conceptual confusion:

**Traditional spectral-correlation OFDR**: This is the original scheme first proposed by Eickhoff and Ulrich in 1981 [1] and further developed by Froggatt et al. in 1998 [7]. It relies on the Fourier transform of the backscattered signal as a function of laser frequency to resolve the fiber trace and retrieves distributed temperature and strain by tracking the spectral shift of local RBS profiles via cross-correlation between reference and measurement states. Characterized by ultra-high spatial resolution down to sub-millimeter scale, it is primarily used for quasi-static measurements. Devices such as the Luna OBR 4600 are based on this principle.

**FMCW-OFDR**: Based on the FMCW technique, this scheme employs a symmetrical sawtooth frequency sweep pattern and extracts beat signals from partitioned spectral sub-bands through parallel processing. While still using RBS as the distributed sensing signature, it achieves significantly higher scan rates at shorter measurement ranges, typically with a corresponding trade-off in spatial resolution. This performance balance makes it particularly suitable for short-to-medium range dynamic monitoring applications.

**φ-OFDR**: Unlike the above two schemes that rely on spectral shift demodulation, φ-OFDR exploits the phase information of the coherent beat signal using I/Q demodulation or Hilbert transform. It provides ultra-high sensitivity to fast dynamic perturbations and constitutes the technical basis for distributed acoustic sensing (DAS) applications.

### 2.2. Error Analysis

The signal model above is based on three ideal assumptions including strictly linear frequency sweep, perfect optical coherence and absence of environmental disturbances. In practical OFDR systems, various non-ideal factors will induce signal waveform distortion and error accumulation, which directly degrade sensing accuracy, effective spatial resolution and long-term measurement reliability. From the generation mechanism, dominant error sources can be classified into three main categories, forming the core motivation for the performance enhancement techniques discussed in subsequent sections.

Sweep non-linearity and sweep repeatability errors constitute the most fundamental error sources in OFDR. As introduced in Section 2.1, TLS sweep non-linearity breaks the uniform sampling requirement on the optical frequency axis, while random sweep span fluctuations across different periods cause distance-axis scale mismatch between reference and measurement states. These issues directly introduce spectral shift estimation bias and spatial drift during cross-correlation demodulation, severely degrading strain and temperature measurement accuracy.

Phase noise and coherence degradation are key factors limiting long-range and high-accuracy measurements. Phase noise mainly originates from the random phase fluctuations caused by spontaneous emission inside the laser cavity, while fiber chromatic dispersion introduces additional phase distortion and amplifies the adverse effects of sweep errors. The impact of phase noise exhibits a significant cumulative effect. As sensing distance increases, the propagation delay difference between the LO and RBS signal increases continuously, resulting in the reduction in their mutual coherence. This leads to attenuation of distance-domain signal peaks, elevated system noise floor and reduced detectability of weak RBS signals. In the demodulation process, phase noise distorts the Rayleigh spectral fingerprint, increases spectral shift estimation uncertainty and ultimately reduces system measurement sensitivity.

Signal interference, spurious peak generation and polarization fading errors mainly originate from amplitude noise, polarization state mismatch and multi-path reflections in the optical link. Amplitude noise mainly includes laser relative intensity noise and parasitic amplitude modulation from optical components, which reduce system SNR and limit spurious-free dynamic range. Owing to the coherent detection nature of OFDR, interference visibility is highly sensitive to the polarization states of reference and RBS light. Random polarization fluctuations during transmission will lead to polarization fading, manifested as an abrupt drop or even complete loss of effective signal intensity. To address this issue, polarization diversity detection (PDD) is widely adopted in practical systems, where a polarization beam splitter decomposes the signal light into two orthogonal polarization components for separate detection and subsequent recombination, thereby effectively mitigating polarization fading. Alternatively, polarization scrambling or the utilization of polarization-maintaining fibers can also be employed depending on specific application requirements. In addition, internal reflections inside the AUX and parasitic reflections from optical connectors and couplers will introduce additional interference paths, generating ghost peaks in the distance domain. Ghost peaks adjacent to true signals will corrupt sliding window demodulation, leading to false alarms or localization deviation.

Based on the above classification of core error sources, the next section systematically reviews representative research advances in OFDR performance enhancement over the past five years, corresponding to the error mitigation requirements identified here.

## 3. Key Technologies for OFDR Performance Enhancement

Numerous studies over the past five years have proposed improvement schemes covering sweep calibration, noise suppression, demodulation algorithm optimization and the co-design of software and hardware acceleration. Based on the focus of the existing literature on specific error sources and corresponding improvement pathways, this section summarizes seven categories of key technologies for OFDR performance enhancement. These categories include sweep non-linearity correction, phase-noise suppression, signal denoising and enhancement, demodulation algorithm optimization, computational efficiency improvement, system hardware optimization and compensation of special effects.

### 3.1. Sweep Non-Linearity Correction

Sweep non-linearity correction is the core prerequisite for high-accuracy OFDR demodulation. Early research on sweep non-linearity correction mainly focuses on software resampling and phase modeling. Guo et al. [8] adopted equal-frequency resampling for sweep non-linearity suppression. The authors extract instantaneous frequency via Hilbert transform and use high-order Taylor terms to correct the instantaneous frequency distribution. This scheme achieves 12.1 µm physical spatial resolution with a 130 nm sweep range and realizes distributed temperature sensing with 1 cm spatial resolution and ±0.15 °C measurement uncertainty over a 105 m fiber link. In 2022, Guo et al. [9] further investigated the joint effects of non-linear sweep noise and random sweep-span bias based on the above framework. The method adds a sweep-span estimation step for each scanning cycle to calibrate random span deviation. It achieves strain measurement accuracy of ±0.51 με, ±5.89 με and ±19.31 με at spatial resolutions of 5 mm, 1 mm and 0.5 mm, respectively. Nevertheless, the two schemes place stringent requirements on DAQ and laser sweep parameters and rely on heavy data processing, which limits their real-time operation capability and general deployability.

**Figure 5:**
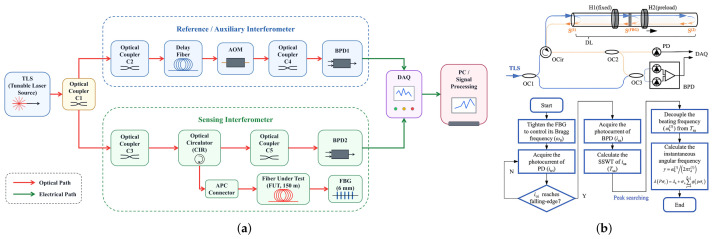
(**a**) Author-drawn modular schematic of an AOM-assisted auxiliary interferometer for non-linear frequency-sweep correction in OFDR. The AOM introduces a fixed frequency shift to increase the beat-signal zero-crossing rate and improve resampling accuracy under short delay-fiber conditions [10]. (**b**) Schematic diagram of the dynamic wavelength calibration method. The upper panel shows the hardware connection diagram, and the lower panel presents the flow chart of the calibration process. In the upper panel, the blue and orange arrows indicate the forward optical path and the backscattered signal path, respectively. The abbreviation H stands for fiber holder. Reprinted with permission from Ref. [11] © Optica Publishing Group.

To alleviate the trade-off between the delay length of AUX and the effectiveness of non-linearity correction, Wang et al. [10] proposed an acousto-optic modulator (AOM) integrated scheme in 2022, as shown in Figure 5a. The method introduces a fixed frequency shift via AOM to increase the beat signal zero-crossing rate, enabling accurate non-linearity extraction even with a short delay fiber. The scheme further facilitates resampling correction and enhances the physical spatial resolution of the sensing system. For high-precision wavelength calibration, Dang et al. [11] adopted the synchrosqueezed wavelet transform (SSWT) to extract high-precision time–frequency trajectories of interference beat signals. The system diagram and calibration flow chart are illustrated in Figure 5b. This method improves the wavelength calibration resolution to 5 fm, increases the spatial resolution from 100 mm to about 1 mm and extends the measurement range to approximately 80 m. However, SSWT has high computational complexity, and an increase in decomposition levels poses a challenge to the processing capability of real-time systems.

For φ-OFDR systems, Liu et al. [12] focused on tuning scanning non-linearity-induced resolution degradation and the susceptibility of phase demodulation to modulation depth variations. The method models non-linear phase noise via polynomial regression and performs digital compensation through matched Fourier transform, with an arctangent function and differential-self-multiplying algorithm to enhance robustness. Simulation results show it achieves approximately 50 cm spatial resolution over a 26.8 km fiber link and 0.1 με minimum strain resolution. However, this study is mainly based on numerical simulation and lacks experimental verification.

Several studies implement sweep linearization at the laser source level. Zhong et al. [13] achieved ultra-linear broadband frequency sweep by combining high-order sideband injection locking with voltage pre-distortion compensation. Using a narrow-linewidth fiber laser and a distributed feedback laser, the scheme realizes a 60 GHz sweep range and 15 THz/s sweep rate with only 17.58 kHz frequency error. And it delivers 5 cm and 7 cm spatial resolution over 1 km and 2 km fiber links, respectively, without complex post-processing. Nevertheless, the effective duty cycle of the laser source is only 50%. Zhu et al. [14] employed a dual electro-optic frequency comb (EOFC) to implement frequency synthesis for broadening the effective sweep range, achieving an equivalent sweep range of 143 GHz and a physical spatial resolution of 0.9263 mm. However, such methods suffer from complex structure and high cost.

Chen et al. [15,16] proposed a synchronous equal frequency resampling (SEFR) scheme to address sensing point misalignment caused by random laser frequency sweep range (LFSR) variations. Specifically, a new linear-frequency sequence is constructed from the common frequency band of scanning and measurement stages, and both are synchronously resampled to simultaneously compensate for non-linearity and point misregistration. It reduces strain measurement root mean square error (RMSE) by up to 41 times and extends the sensing distance to 70 m. The main trade-off of this scheme is a certain degree of degradation in spatial resolution.

Furthermore, Liu et al. [17] proposed an adaptive sweep non-linearity compensation method based on phase prediction in 2024. The method uses a short-delay interferometer to obtain TLS instantaneous optical frequency and synthesizes beat signals with different effective delays for full-range optimization. It achieves 0.6 mm physical spatial resolution over 100 m with a 20 m delay fiber, while high computational complexity reduces its real-time processing capability.

Table 1 summarizes the main methods and core performance of all the above schemes. Collectively, these sweep non-linearity correction methods represent a spectrum of trade-offs between computational complexity, correction accuracy, hardware overhead and applicable sensing ranges. Software-based approaches such as Hilbert transform-based resampling and SSWT achieve high correction accuracy without hardware modifications but suffer from heavy computational burdens, making them particularly suitable for offline or laboratory-oriented high-accuracy measurements. In particular, SSWT provides an exceptional wavelength calibration resolution of 5 fm but requires significant computational resources that limit real-time implementation. Hardware-assisted methods like AOM-aided resampling and dual EOFC frequency synthesis achieve a better balance between accuracy and speed but introduce additional optical components and system complexity. Source-level linearization techniques such as high-order sideband injection locking eliminate the need for post-processing entirely but are currently limited by low laser duty cycles and high system costs. For practical engineering applications, the SEFR scheme offers a viable compromise by simultaneously correcting non-linearity and point misregistration with moderate computational overhead, albeit with a slight degradation in spatial resolution.

### 3.2. Phase-Noise Suppression

In long-range, high-accuracy OFDR systems, accumulated phase noise is the dominant limiting factor that severely degrades overall measurement performance. Existing phase-noise suppression techniques can be broadly classified into two primary categories, algorithmic compensation and hardware matching. Algorithmic compensation focuses on the modeling, estimation and cancellation of the phase-noise component in the received signal. Hardware matching improves compensation efficiency and reduces residual phase noise via dedicated system architecture design or optimized delay configuration.

**Figure 6:**
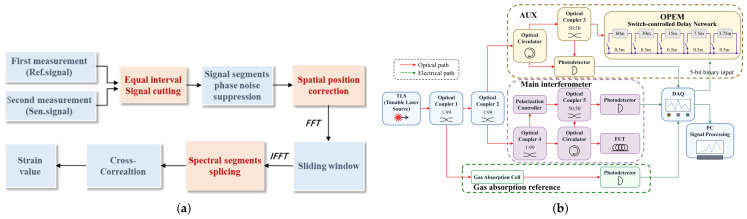
(**a**) Flowchart of the spectral-splicing strain-demodulation procedure: the long sweep is segmented, each segment is processed by the PPNE-deskew filter, spatial positions are corrected and the segments are stitched in the frequency domain to recover a wideband spectrum. Reprinted with permission from Ref. [19] © Optica Publishing Group. (**b**) Author-drawn modular schematic of an OPEM-assisted OFDR system with a gas absorption reference and main interferometer. Segmented measurements with optimal delay matching are stitched to obtain full-range strain distribution [22].

For algorithmic compensation schemes, Zou et al. [18] developed a periodic-phase-noise-estimated deskew filter (PPNE-deskew filter) method. They use moving-average filtering to suppress differentiation-induced noise amplification, third-order Taylor expansion for periodic phase noise estimation and a deskew filter to eliminate beat noise along the FUT. Experimental results show that this scheme achieves a physical spatial resolution of 535 µm over an 8 km fiber link with a range-resolution^−1^ product (RRP) of $1.5\times 10^{7}$, 2.5 times higher than that of high-order optical phase-locked loop (OPLL) schemes. But it introduces a heavy computational burden with a typical 1-min processing time. Building upon the PPNE framework, Zhu et al. [19] further proposed a spectral splicing method (SSM). The flow chart is illustrated in Figure 6a. This method divides the long-sweep signal into independent segments for PPNE-deskew processing and spatial position correction before frequency-domain stitching to recover a highly consistent wide spectrum. It extends the manageable sweep range to 10 nm, reduces segment-induced position errors from the 10 m scale to the millimeter level and achieves 1 cm spatial resolution, ±3.2 με (3σ) strain sensitivity and a 10,000 με measurable strain range over a 1 km fiber link.

For hardware matching-based schemes, Liang et al. [20] optimized the system hardware and proposed a multi-arms interferometer phase noise compensation (MAI-PNC) method. MAI-PNC creates multiple optimal compensation points across the full fiber link via interferometers with different arm length differences and performs segment-wise phase noise compensation followed by distance-domain stitching. This scheme achieves a spatial resolution of 2 cm and a measurable strain range up to 3000 με. The RMSE of demodulation is reduced from 0.00310 nm to 0.00035 nm, and the effective sensing distance is extended from 104 m to 338 m. Along the delay-length matching paradigm, Fu et al. [21] reduced residual phase noise by optimizing the match between the AUX delay fiber and the FUT length, achieving 2 mm resolution and better than 1.5 με (2σ) strain accuracy over a hundred-meter scale. This scheme requires prior knowledge of the FUT length or the ability to adjust the delay fiber length, which limits its applicability in scenarios with unknown or dynamically varying fiber links. Building on a similar idea, Yang et al. [22] further implemented a coded delay fiber module (OPEM) to deliver on-demand optimal delay and obtained full-range strain distribution via segmented measurements and stitching, as shown in Figure 6b. The modular design increases flexibility, and the minimum step size of the OPEM is 3.25 m.

Table 2 also summarizes the key methods and representative results of the above schemes in this section.

### 3.3. Signal Denoising and Enhancement

Signal denoising and enhancement techniques aim to suppress random noise, spurious peaks, multi-peak interference and sidelobe-induced ghost peaks through post-processing without hardware modification. These techniques improve the identifiability of CC results and distance-domain traces. Typical approaches include image-domain denoising with image or system response priors for converted CC matrices and deconvolution or morphological suppression for deterministic artifacts such as sidelobe ghost peaks.

**Figure 7:**
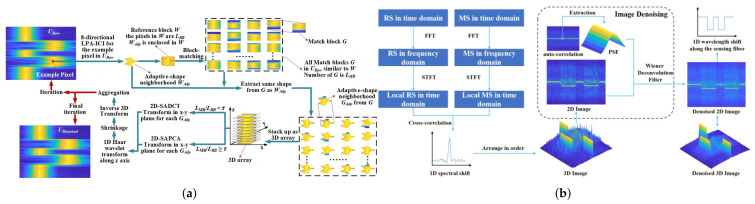
(**a**) Image-domain denoising workflow based on BM3D-SAPCA and empirical Wiener filtering applied to the 2D cross-correlation matrix for long-distance distributed strain sensing. The red arrows indicate the non-iterative decision/output flow, whereas the cyan arrows indicate the iterative denoising loop. Reprinted with permission from Journal of Lightwave Technology, Ref. [25] © 2022 IEEE. (**b**) Wiener-deconvolution processing of the 3D cross-correlation distribution of local RBS spectra. The PSF is estimated from the local-spectrum autocorrelation to recover fine spectral features buried in noise. Reprinted with permission from Journal of Lightwave Technology, Ref. [27] © 2024 IEEE.

Image-domain denoising approaches have been widely developed. Li et al. [23] introduced a method combining distance compensation with image wavelet denoising. They adopt polarization control and polarization-diversity reception to enhance SNR and process the 2D matrix stacked by CC results via 2D wavelet thresholding. This method achieves a spatial resolution of 2.56 mm over a 25 m fiber link, with a measurable strain range of 200 με to 2000 με and ±20 με accuracy. Its wavelet parameter selection depends on specific noise conditions, and its generalization ability for long-distance scenarios requires further validation. Qu et al. [24] adopted a similar image-based CC strategy and used total-variation or Gaussian filtering to suppress noise, achieving 1.3 mm resolution over 52 m fiber and resolving strain steps of 100 με to 500 με. To improve long-range performance, Pan et al. [25] employed iterative denoising based on shape-adaptive principal component analysis block-matching three-dimensional filter (BM3D-SAPCA) with empirical Wiener filtering. The denoising process is shown in Figure 7a. Over a 200 m all grating fiber, this method achieves 5 cm resolution and 2 με strain resolution, with significantly reduced maximum measurement error and standard deviation.

Suppressing deterministic artifacts and sidelobe-induced ghost peaks is another major challenge. Guo et al. [26] addressed PSF sidelobes caused by TLS spectral impurity and internal reflections in the AUX. This method extracts PSF from AUX signal to construct a Wiener deconvolution filter and applies a second-stage deconvolution to the main interferometer signal for ghost peak removal. Experiments with a low-cost distributed feedback (DFB) TLS and a 26 m test length demonstrate a cleaner distance trace after processing. Lou et al. [27] also adopted Wiener deconvolution and emphasized that the autocorrelation function of the local Rayleigh spectrum can serve as a theoretical basis for PSF, as shown in Figure 7b. They performed frequency-domain deconvolution on the CC image and recovered events buried in noise at 200 µm spatial resolution, outperforming Gaussian filtering. A comparative analysis shows that the scheme in [26] mainly targets deterministic ghost peaks from system sidelobes, while the work in [27] focuses more on recovering fine details obscured by random noise in ultra-high-resolution sensing conditions.

For high-resolution temperature demodulation scenarios, Li et al. [28] proposed an adaptive morphological processing (AMP) method to address increased bad points and spurious peaks in measurement results through constructing multiple candidate morphological opening operations and fusing them with weights determined by a difference metric. Under a 40 m polyimide (PI)-coated fiber with a 2 mm gauge length at the distal end, the method reliably reconstructs temperature gradients ranging from 43 °C to 55 °C. The gauge length accuracy is increased by about 3 times with 0.563 s processing time. For humidity sensing applications, Bai et al. [29] combined PI-coated fiber with an adaptive 2D bilateral processing approach. By weighting both spatial distance and intensity similarity, the method denoises while preserving gradient edges. Over a 48 m fiber link, it achieves a spatial resolution of 4 mm with a peak-to-peak measurement error of 3.1 pm, approximately half of that obtained by conventional processing.

Table 3 summarizes representative studies on signal denoising and enhancement.

### 3.4. Demodulation Algorithm Optimization

Demodulation algorithm optimization aims to achieve a better balance among spatial resolution, measurement range, accuracy, robustness and real-time capability. Different from pure signal denoising techniques, this research direction mainly exploits structural priors of the demodulation process to suppress spectral mismatch, spurious peaks and phase jumps induced by large strain. Novel estimation strategies are also developed to extend the dynamic measurement range or improve the spatial resolution.

**Figure 8:**
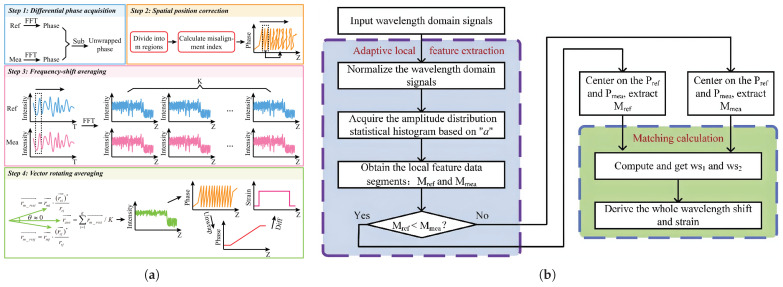
(**a**) Principle of coherent-fading suppression in φ-OFDR using FSAV and RVS, combined with spatial position correction to improve measurement range and accuracy. Reprinted with permission from Ref. [35] © Optica Publishing Group. (**b**) Operation flowchart of the ALFEM algorithm. It demonstrates the full strain demodulation workflow for the BEOF-based OFDR system. Reprinted with permission from Ref. [36] © Optica Publishing Group.

Some studies focus on φ-OFDR systems. Zhao et al. [30] proposed a robust phase unwrapping and serial phase correction procedure with density distribution denoising to address deformation measurement failures caused by phase noise, decoherence and misalignment of scattering units. This scheme achieves deformation measurement with a physical spatial resolution of 40 µm. Guo et al. [31] proposed a statistical-distribution-based phase-jump filtering method to suppress unwrapping jumps induced by quadrant discrimination errors and random phase noise. In a 30 m fiber Bragg grating (FBG)-array system, this method achieves 1.76 με strain accuracy at 20 mm spatial resolution with a φ-OFDR computation time only 3.2% of that of coherent OFDR. But intrinsic phase noise remains a severe limiting factor for micrometer-level resolution or long-range sensing applications. In large-strain sensing scenarios with severe unwrapping errors in low-SNR regions, Yang et al. [32] established a phase-prediction-assisted demodulation algorithm. They use a polynomial fit on high-SNR segments to predict the phase trend in low-SNR segments and then compensate phase jumps and recover outliers via interpolation. Experiments demonstrate measurement over 0–2500 με at 1 mm resolution with 17.32 με measurement error and a processing time of 0.063 s for a 0.8 m sensing length. Liu et al. [33] combined complex-domain denoising with segmented spatial position correction and employed high numerical aperture fiber (HNAF) to enhance backscatter, achieving 0.89 mm resolution, 1.5 με RMSE and a maximum strain of 2050 με. But this approach depends on HNAF and requires manual tuning of denoising parameters. Liu et al. [34] further proposed a general adaptive phase-noise suppression scheme based on continuity conditions at adjacent-window boundaries. The scheme triggers phase compensation when the difference between adjacent-window mean values approaches 2nπ, reconstructs high-noise windows and applies wavelet packet denoising. At 1.5 mm sensing resolution, it achieves 0.9343 με strain accuracy over an effective range of 20–270 με. As coherent fading remains a dominant source of unwrapping anomalies in high-resolution sensing, Li et al. [35] enhanced system robustness via a combined scheme of frequency-shift averaging (FSAV), rotating vector summation (RVS) and position correction. The fading noise suppression principle of the method is shown in Figure 8a. The scheme achieves a measurement range of 2000 με at 0.54 mm spatial resolution, with 0.87% measurement accuracy and a relative standard deviation of 0.42% in a 3 h long-term stability test.

OFDR systems are prone to spectral mismatch under large strain conditions, and the measurement reliability needs to be further improved. Qu et al. [37] proposed a time-optimized interpolation and distance-domain compensation scheme by considering the coupling among processing speed, spatial resolution and strain measurement range. By compressing the zero-padding scale and iteratively compensating for position bias induced by large strain using the Rayleigh scattering centroid, they achieve 2 mm resolution and 10,000 με strain range over an 11.2 m fiber. And the processing time is reduced by 76 times compared with conventional methods. The sensing distance is relatively short, and the computational performance in long-range scenarios remains to be verified. Fu et al. [38] proposed the adaptive zero padding (AZP) and distance compensation method (DCM) under exposed single mode fiber (E-SMF) conditions. At 1.5 mm resolution, the maximum measurable strain is extended to 9000 με, while computation time is reduced from 138.842 s to 0.476 s. To address fake-peak errors at strain area edges, Liang et al. [39] proposed a local spectrum division method. Unreliable segments are screened using a quality factor and iteratively re-demodulated, and the final estimate is obtained via weighted fusion. Over a 15 m fiber link, strains up to 1900 με are measured, and RMSE is reduced from 0.00878 nm to 0.00026 nm. Li et al. [40] proposed a self-correcting 2D CC approach for large-strain measurement. In the frequency domain, a 2D array of reference signals with different assumed position deviations is constructed and cross-correlated with the measurement signal to obtain similarity. A self-correcting dictionary based on standard deviation is used to enhance main-peak identification. At the end of a 50 m fiber, strain gradients of 1000–8000 με are measured with 3 mm resolution, reducing mean absolute error from 14.12 pm to 6.12 pm. Zou et al. [41] proposed the spectral shift adjacent point difference method to mitigate spurious peaks. This method obtains an initial drift baseline from two unstrained scans, removes false peaks via differential thresholding and fills missing points by interpolation. A strain range of 0–10,800 με is achieved over a 20 m fiber with ±43.6 με accuracy. The spatial resolution is 7.84 mm, about half that of conventional methods.

Aiming at spurious peaks induced by unequal amplitude weighting in CC computation, Liu et al. [42] designed a spectral segmented normalized CC (SN-CC) scheme. This method segments the local spectrum according to its free spectral range and normalizes each segment before CC. At 3000 με strain, the method achieves 3.2 mm spatial resolution, providing a 5.6 times improvement over the conventional approach. Zheng et al. [43] proposed a modified longest common substring (LCS) algorithm, mapping a 1D spectral sequence to a 2D similarity image and formulating demodulation as a line-detection problem. Experimental results show an approximately 18.7 times improvement in strain measurement range relative to normalized CC (NCC).

In terms of improving resolution via time–frequency analysis, Liang et al. [44] replaced the short-time Fourier transform (STFT) with the Margenau Hill spectrogram (MHS) to achieve better time–frequency concentration. The spatial resolution is improved from 5.0 mm to 0.1 mm while maintaining a sensing quality factor of 0.515. The computational complexity of MHS is significantly higher than that of STFT. Liang et al. [45] subsequently extended their idea to Cohen’s class methods and proposed Cohen’s class time–frequency analysis with a modified kernel function. Over a 14 m sensing distance, the spatial resolution is improved from 6.05 mm to 0.16 mm, and an RMSE of $2.91\times 10^{-3}$ nm is achieved over a 0–1000 με range. But the kernel design relies on empirical tuning. To improve the dynamic response capability of the system, Lin et al. [46] proposed spectral vernier OFDR, decoupling measurement range from frequency response. This scheme uses a low-speed wideband scan as a coarse main scale and a high-speed narrowband sampling as a vernier and performs demodulation by tracking CC peak drift. The scheme achieves a strain range of 10,856 με and a 1.21 kHz bandwidth at 10 cm spatial resolution. However, dynamic experiments only demonstrated low-amplitude and low-frequency vibration. And the approach relies on an optical switch to toggle two modulation modes, so switching synchronization may affect real-time performance. Finally, Lv et al. [36] combined backscattering enhanced optical fiber (BEOF) with adaptive local feature extraction and matching (ALFEM) to simultaneously improve strain range and demodulation speed. The operation flowchart of the algorithm is shown in Figure 8b. BEOF enhances RBS by about 20 dB using an ultra-weak grating array, and ALFEM extracts only high-amplitude features for matching to reduce computation load. Experimental results show that the scheme achieves 400 µm spatial resolution, 4800 με strain range and 1.7 με strain resolution. The demodulation time is reduced to approximately 25% of that of the conventional method, yet the scheme is only validated on a relatively short 22.95 m fiber link.

The key methods and main results of the above schemes in this section are summarized in Table 4.

### 3.5. Computational Efficiency Improvement

Computational efficiency improvement aims to overcome the real-time bottleneck of OFDR in dynamic monitoring and deployment. Existing research in this field mainly follows two complementary technical routes. The first route focuses on processing acceleration via parallel hardware platforms such as graphics processing units (GPUs) and field-programmable gate arrays (FPGAs). And the other route aims to reduce redundant computations through strained region screening and novel estimation strategies that replace dense interpolation operations.

Regarding hardware acceleration, Wang et al. [47] systematically refactored demodulation steps such as FFT, zero padding and CC into GPU-parallel kernels and adopted double-buffered memory management to realize pipelined integration of data acquisition and real-time processing. The processing speed is improved by approximately 81 times relative to CPU, enabling 60 Hz real-time dynamic strain sensing over a 200 m fiber link at 20 cm resolution. Liang et al. [48] proposed a fully FPGA-integrated scheme, implementing data acquisition, 2D-FFT, sliding window and CC in parallel on a single FPGA. Compared with CPU, the total processing time is reduced from 1436 ms to 67 ms and achieves a strain standard deviation of 3.49 με at approximately 0.28 mm spatial resolution. But the validated sensing length of the system is only 25 m. For multi-core fiber (MCF) shape reconstruction, Shan et al. [49] migrated segmentation, FFT, CC and peak search to GPU-parallel execution, reducing the reconstruction time by nearly 21 times for 2D and 3D shapes. At 5 mm resolution, the maximum reconstruction errors are 3.23% and 2.47% for 2D and 3D, respectively.

**Figure 9:**
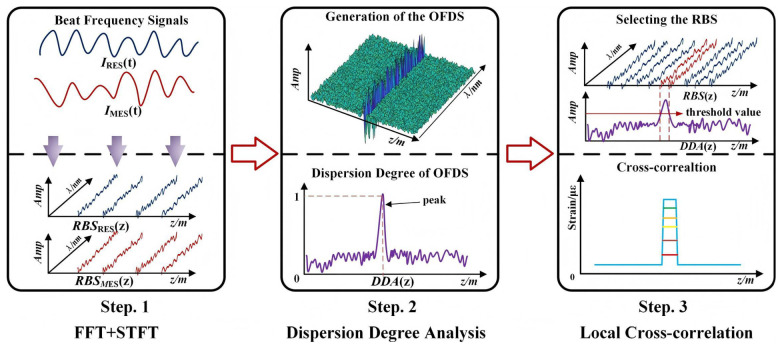
Flowchart of DDA-based OFDR demodulation method: differential optical-frequency-domain spectra (OFDS) are computed along the fiber, a variance threshold identifies strained segments and localized cross-correlation is performed only on selected regions to accelerate demodulation. Reprinted with permission from IEEE Sensors Journal, Ref. [51] © 2023 IEEE.

In terms of algorithm simplification, Bai et al. [50] put forward a demodulation scheme combining wavelength domain differential accumulation (WDDA) and local CC (LCC). WDDA is used to quickly localize strained segments, and CC is then computed only for the identified region. This scheme achieves a speedup ratio of 5.3 to 6.4 times and can effectively detect strain larger than 10 με. However, its efficiency advantage is only prominent in scenarios with localized strain distribution. Zhang et al. [51] proposed dispersion degree analysis (DDA), as shown in Figure 9. A variance threshold on differential spectra is used to identify strained regions, and LCC is performed only on the selected segments. For a 40 m fiber link at 8 mm spatial resolution, the demodulation efficiency is improved by approximately 7.18 times, with a minimum detectable strain of 7.81 με. Dense interpolation in peak estimation constitutes another major computational overhead. For this high-overhead processing link, Zhang et al. [52,53] designed an enhanced Buneman frequency estimator (BFE). This estimator directly calculates the peak offset from a coarsely sampled CC spectrum, which completely avoids dense interpolation operations and improves the demodulation efficiency by more than 17 times. Its estimation performance depends on the approximation degree between the actual CC spectrum and the ideal sinc function.

Table 5 summarizes the above methods.

### 3.6. System Hardware Optimization

System hardware optimization improves OFDR performance fundamentally through innovations in optical materials, structures and sensing formats, targeting backscatter enhancement, complexity reduction and cost control. Unlike algorithmic schemes, it breaks physical-layer bottlenecks to form repeatable engineering modules.

Several hardware optimization efforts focus on enhancing RBS. Wang et al. [54] employed femtosecond laser inscription to enhance RBS by more than 40 dB. Using a low-cost TLS with only 1 nm tuning range combined with corresponding signal processing, they achieve 4.8 cm resolution and an RMSE of less than 2.70 με. Nevertheless, excessive backscatter enhancement introduces high optical loss and limits the maximum sensing length, and the scheme is only validated in a narrow strain range of 10–50 με. Du et al. [55] fabricated a Rayleigh-scattering-enhanced SMF (i.e., E-SMF) via UV exposure, achieving a 37.3 dB increase in Rayleigh scattering enhancement and successfully demodulating 200–2600 με strains at 2.0 mm resolution. Chen et al. [56] developed an OFDR system integrated with a weak-reflection fiber Bragg grating sensors (WRFBGs), whose RBS intensity is about 25 dB stronger than those of standard fiber, significantly improving SNR and accuracy. Direct peak searching is used instead of CC to enable a 38 Hz response speed. This scheme successfully captures the yield point position in a rebar tensile test and realizes effective monitoring of strain steps exceeding 25,000 με.

Simplifying system structure and reducing cost is another important objective. Zhong et al. [57] proposed a single-interferometer self-compensation method (SCM). The FUT end face is shaped into an arc to produce a stable reflection signal that replaces the AUX. Spatial resolutions of 3 mm and 5 mm are achieved over 108 m and 170 m sensing distances, respectively, with a temperature sensitivity of 1.34 GHz/°C. However, the consistency of end-face reflection fabrication is highly process-dependent. Belokrylov et al. [58] combined the AUX and gas absorption cell into a single channel to simplify hardware and reduce cost and decoupled signals via filtering or empirical mode decomposition (EMD). On a fiber of about 50 m, filtering and EMD achieve precision of 0.106 mm and 0.119 mm, respectively. But the frequency filtering requires high-order filters and takes more than 40 s per computation, resulting in poor real-time performance. The EMD-based method requires manual selection of intrinsic mode functions (IMFs) and lacks an automatic adaptation mechanism.

From the sampling mechanism perspective, Jin et al. [59] proposed a compressive sensing-based scheme. Two unbalanced AUXs are used to generate coprime non-uniform sampling sequences, and sparse reconstruction recovers the signal at a sampling rate below the Nyquist limit. This approach reduces the number of zero-crossing points by an order of magnitude compared with conventional schemes and achieves a 200 m sensing distance with 1 mm spatial resolution. The main limitations include the need for post-processing for high-order phase-noise compensation, limited real-time capability and reliance on spatially sparse structures such as FBGs, restricting applicability.

**Figure 10:**
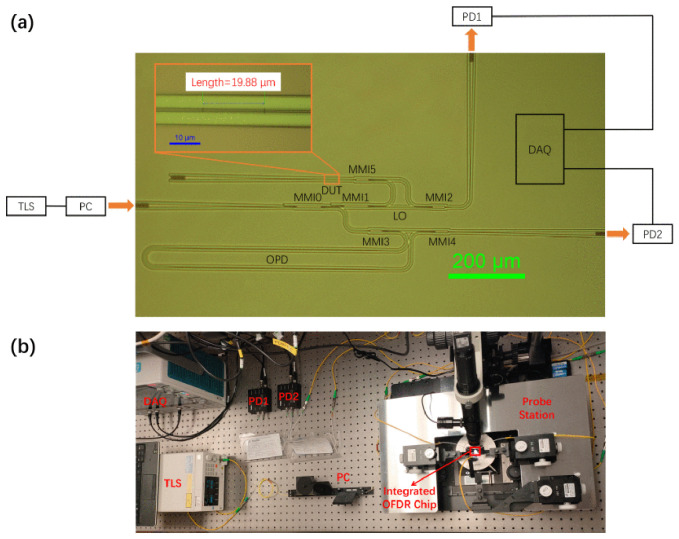
Experimental setup of integrated OFDR characterization. Reprinted with permission from Journal of Lightwave Technology, Ref. [60] © 2024 IEEE. (**a**) The schematic diagram. (**b**) The deployment. The integrated OFDR chip is placed on the self-designed three-dimensional adjustable probe station.

Integrated OFDR is emerging as a key direction for reducing size, weight and cost while enhancing deployability. In 2024, Han et al. [60] reported the first silicon-on-insulator (SOI)-based integrated OFDR system for continuous distributed measurements using RBS. The system uses a multi-mode interference (MMI) coupler for power splitting and circulator-like functions and adopts heterodyne interferometric detection for signal acquisition. The schematic diagram and deployment are shown in Figure 10. Experiments achieve an 8.28 µm physical spatial resolution over a 43 nm wavelength sweep, close to the theoretical limit of 7.51 με. And the chip has a footprint of approximately 1.1 mm × 0.5 mm, demonstrating the feasibility of high-resolution OFDR system miniaturization. This silicon photonic scheme shows broad application potential in multiple industrial scenarios, benefiting from its high integration density, low power consumption and complementary metal-oxide-semiconductor (CMOS) compatibility. Nevertheless, on-chip coupling and insertion losses lead to lower SNR compared with conventional systems, and further performance optimization is still required.

However, chip-scale OFDR systems still exhibit lower SNR, reduced dynamic range and shorter practical sensing distance compared with conventional fiber-based setups. This limitation mainly arises from propagation losses in silicon waveguides and non-negligible insertion losses from grating couplers and other passive components, which collectively weaken the detectability of low-level RBS. In addition, on-chip waveguide dispersion may distort the frequency-to-distance mapping and broaden the PSF, especially for broader wavelength sweeps or longer on-chip sensing paths. Addressing these bottlenecks through low-loss waveguide and coupling designs, dispersion-aware calibration, backscattering enhancement and higher-level monolithic or heterogeneous integration therefore remains a core challenge for next-generation integrated OFDR systems.

Table 6 summarizes the key methods and main results of the above schemes in this section.

### 3.7. Compensation of Special Effects

In long-range OFDR sensing and applications based on special optical fibers or photonic devices, including polarization-maintaining fiber (PMF) and reduced-cladding single-mode fiber (RC-SMF), non-ideal effects such as polarization fading, dispersion mismatch and accumulated birefringence become dominant performance-limiting factors. These effects can severely degrade correlation peak quality and spatial resolution and even induce inter-axis misregistration and complete demodulation failure.

Xie et al. [61] improved distributed birefringence measurement in PMF. They calculate distributed birefringence by demodulating the Rayleigh spectrum shift difference between the fast and slow axes of PMF. This scheme achieves a spatial resolution of 5 cm and a measurement uncertainty of $6.8\times 10^{-7}$ over a 2257 m fiber link and enables effective identification of winding process defects in fiber coils. To address inter-axis positional mismatch induced by accumulated birefringence in long PMFs, Lin et al. [62] proposed a dynamic birefringence delay correction scheme. Coarse pre-correction and point-by-point fine compensation are combined to dynamically adjust window positions so that the S and P axes remain aligned. Experiments achieve 10 cm resolution and $1.4\times 10^{-7}$ accuracy over a 5020 m PMF link with only 1 s per sweep, enabling fine diagnostics of fiber gyroscope coils. The stability of the scheme may still be affected by fiber inhomogeneities and residual phase noise.

Zou et al. [63] proposed a distributed chromatic dispersion compensation scheme based on a mismatch factor. The mismatch is quantified by polynomial fitting of the quadratic phase term, and a dispersion phase error signal is constructed to cancel dispersion errors segment by segment. For a 500 m RC SMF link, the physical spatial resolution is improved from 5.9 mm to 40.7 µm, and a physical resolution better than 15 µm is achieved in internal reflection analysis of a multi-functional integrated optical chip. For polarization fading, Mou et al. [64] proposed a hybrid-polarization diversity scheme. This method inserts a 45° polarizer in the reference path and adopts a PMF-based structure to convert random polarization fluctuations into predictable frequency-domain shifts for subsequent compensation. Over a wide wavelength tuning range from 1480 nm to 1640 nm, polarization angle fluctuations are reduced from 40.93° to 2.81°, with an ultra-high physical spatial resolution of 6 µm and a sensitivity of −145 dB.

The key methods and main results of the above schemes are summarized in Table 7.

## 4. Applications of OFDR

As introduced in Section 2.1, OFDR technology has evolved into different operating/demodulation modes with distinct performance characteristics and application scenarios. In this section, we systematically review the diverse engineering applications of OFDR. Unless otherwise specified, the discussed systems refer to traditional spectral-correlation OFDR or FMCW-OFDR. Given the relatively limited number of studies based on φ-OFDR and its distinct demodulation principle, we will explicitly mark φ-OFDR-based systems when they appear to avoid conceptual confusion.

### 4.1. Temperature, Humidity and Strain Sensing

RBS-based OFDR provides high-spatial-resolution distributed measurements with intrinsic high sensitivity to temperature, humidity and strain. Recent relevant research mainly focuses on three key areas: advances in measurement range and accuracy, temperature/humidity multi-parameter decoupling and SHM.

**Figure 11:**
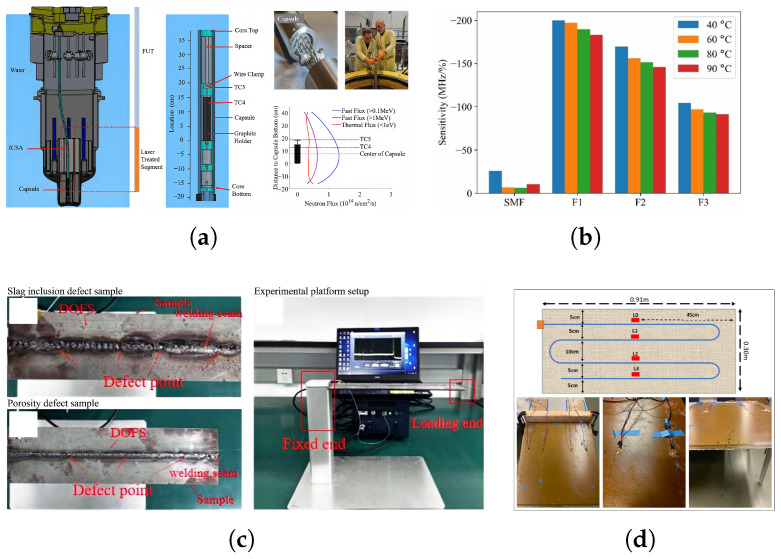
Representative system architectures and engineering applications of OFDR technology. (**a**) Deployment of fiber optic sensors for temperature profiling under high temperature of 560 °C and intense neutron irradiation. The leftmost panel shows the 3-D cutaway model of the MITR, the middle panel shows a zoomed-in view of the assembly, the two upper-right photographs show the capsule cross-section and fiber sensor installation process, and the lower-right plot shows the neutron-flux distribution in the ICSA facility with the capsule position indicated. Reprinted with permission from Journal of Lightwave Technology, Ref. [66] © 2021 IEEE. (**b**) Temperature-dependent humidity sensitivity of a standard acrylate-coated SMF and three polyimide-coated SMFs: F1 with a 62.5 µm cladding radius, 15 µm coating thickness and 6.4 µm mode-field diameter; F2 with a 40 µm cladding radius, 11 µm coating thickness and 6.4 µm mode-field diameter; and F3 with a 62.5 µm cladding radius, 15 µm coating thickness and 4.2 µm mode-field diameter. Reprinted from [67]. (**c**) OFDR-based weld-defect inspection using surface-bonded distributed optical fiber sensors, including a slag-inclusion defect sample, a porosity defect sample and the experimental platform setup. Reprinted from [71]. (**d**) Embroidery-based distributed fiber optic sensing textile embedded in a composite panel, showing the fiber layout and cantilever test setup. Reprinted from [72].

A key challenge for conventional OFDR is the degradation of distributed sensing performance under extreme high temperature. Li et al. [65] designed a specialized waveform conversion circuit for frequency resampling alongside a moving reference spectral strategy. This setup successfully compensates for laser non-linearity and mitigates high-temperature spectral drift. Additionally, it extends the effective temperature measurement range by 2.9 times while achieving a high spatial resolution of 0.2 mm. Wu et al. [66] explored the limits of OFDR in extreme environments using femtosecond laser-enhanced optical fibers. These fibers withstand 560 °C and intense neutron irradiation, enabling the first in-core temperature profile measurement. The deployment configuration is illustrated in Figure 11a. Despite an improved initial SNR, prolonged operation causes radiation-induced attenuation (RIA) and signal degradation.

The hygroscopic expansion of fiber coatings introduces cross-sensitivity among humidity, temperature and strain. Lu and Schukar [67] analyzed strain transfer in optical fibers with hygroscopic coatings using a conventional 1D model and a 3D analytical model based on Lamé’s equations. Their OFDR-based experiments on polyimide-coated fibers further showed that the humidity sensitivity decreases with increasing temperature, as summarized in Figure 11b, highlighting the temperature dependence of strain-based fiber-optic humidity sensing. Qin et al. [68] exploited PI-coated PMFs to decouple temperature and humidity via orthogonal polarization axes. This method, unfortunately, reduces spatial resolution to 5 cm due to wide spectral sliding windows. Li et al. [69] separated signals using a spatial-domain time-delayed multiplexing topology with delay fibers. This architecture enables multi-channel synchronous acquisition but increases optical path complexity and insertion loss. Feng et al. [70] integrated OFDR with cascaded Fabry–Perot (FP) cavities and digital signal processing algorithms to achieve decoupling with a 41 µm resolution. However, the spectral bandwidth of this quasi-distributed technique inherently limits the number of sensing points.

In geotechnics, Jiang et al. [73] applied OFDR to sandstone uniaxial compression tests to reconstruct radial strain fields. In their study, early fiber breakage post-peak prevented residual strength monitoring. Chen et al. [74] processed strain distributions with an integral algorithm to invert micro-crack widths at the 10 µm level. The result shows that tight-sheath optical fibers (TSSOFs) have strain transmission lag compared to PI-coated sensing optical fibers. In weld-strength inspection, Chen et al. [71] employed OFDR-demodulated distributed optical fiber sensors bonded near welded joints to detect defect-induced strain anomalies, as illustrated in Figure 11c. Their experiments on slag-inclusion and porosity defect samples demonstrated that weld defects could be identified and localized from one-dimensional distributed strain profiles, with a reported spatial precision of 0.64 mm. This method enables non-destructive, high-sensitivity and high-spatial-resolution weld assessment based on surface-bonded distributed strain measurements. Zhao et al. [75] proposed combining OFDR with the inverse finite element method (iFEM). They applied this combined approach to identify loads on wing panels. Zhang et al. [76] and Lin et al. [77] employed OFDR to investigate the thermal strain and thermal bias drift of km-level fiber optic gyroscope (FOG) coils. By optimizing the pigtail length, Zhang et al. achieved over 60% reduction in the thermal bias drift of FOG coils. Additionally, Lin et al. corrected the spectral position deviation to obtain high-precision strain data of long-distance FOG coils. OFDR has also been widely implemented in flexible distributed sensing platforms. Biondi et al. [72] developed a distributed fiber optic sensing textile (DFOST) by embroidering a polyimide-coated optical fiber into a textile substrate and embedding it into a composite panel, as illustrated in Figure 11d. Interrogated by OFDR, the embedded textile sensor captured both static loading and dynamic vibration responses in the composite cantilever test, with a reported spatial resolution of 2 mm and a minimum strain resolution of 10 με. Katrenova et al. [78] constructed a 2D pressure sensing map by embedding SMF into silicone. The system achieves high-resolution distributed pressure measurement and calibrates the pressure sensitivity coefficient, demonstrating good feasibility in biomedical applications such as bite force detection.

### 4.2. Acoustic and Vibration Sensing

By virtue of its excellent spatial resolution and high SNR, OFDR exhibits enormous application potential in the fields of DAS and vibration monitoring. The research work of this subsection will focus on key technical directions including ultra-long-range system architecture design, system simplification based on internal modulation technology, high-precision vibration demodulation algorithm optimization and sensing performance enhancement via novel light source selection.

For the core demand of ultra-long-range vibration monitoring, significant advancements have been made in OFDR system architectures and demodulation algorithms. Qin et al. [79] proposed a dual-ended unrepeated OFDR system for ultra-long-range vibration monitoring, which utilized remote-pumped erbium-doped fiber amplifier based on remote pump light source. A segmented CC algorithm is adopted for vibration localization. The single-ended subsystem is experimentally validated to operate up to 222 km, whereas the dual-ended system is designed to push the sensing range to 400 km.

**Figure 12:**
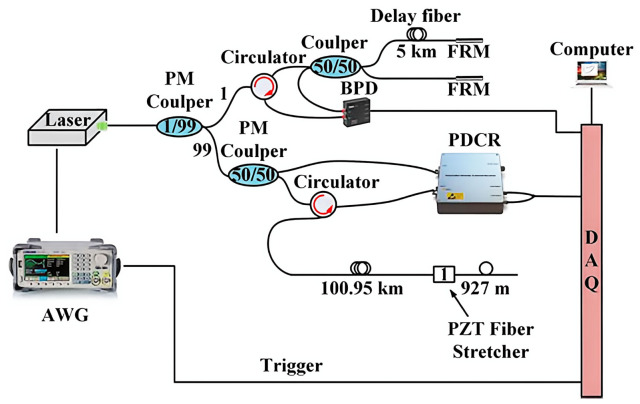
Configuration of the internally modulated OFDR system for long-range distributed vibration sensing, using an AWG-controlled silicon-based photonic-chip laser to achieve optical-frequency sweeping and reduce the complexity of conventional external-modulation architectures. Reprinted from [80].

Focusing on system complexity reduction, internal modulation technology has become a key research direction for OFDR vibration sensing, with multiple optimized schemes verified in engineering. Yue et al. [80] proposed a long-range distributed vibration sensing system based on internally modulated OFDR, as shown in Figure 12. By using an AWG-controlled silicon photonic-chip laser for optical-frequency sweeping, the system reduced the complexity of conventional external-modulation OFDR and localized a vibration event at approximately 100.95 km with a sensing spatial resolution of 16.8 m. Guo et al. [81] developed an amplifier-free single-ended internal-modulation OFDR system for ultra-long-range distributed vibration sensing. They compensated for the inherent frequency tuning non-linearity of internal modulation and innovatively combined the Pearson correlation coefficient (Pearson-CC) with the pruned exact linear time (PELT) algorithm for vibration demodulation. This adjustment secures an amplifier-free 100.9 km range with an 868 pε strain resolution. Furthermore, it demonstrates high linearity ($R^{2}$ = 0.9993) between the differential Pearson-CC and vibration amplitude.

High-frequency vibrations induce time-variant phase modulation in OFDR measurements. Li et al. [82] designed an adaptive 2D-CC algorithm to estimate and compensate for instantaneous beat frequency shifts induced by high-frequency vibrations. This method successfully detects vibration signals up to 18 kHz on a 100 m fiber, maintaining a 12 cm spatial resolution and improving crosstalk suppression by 40.5 dB.

**Figure 13:**
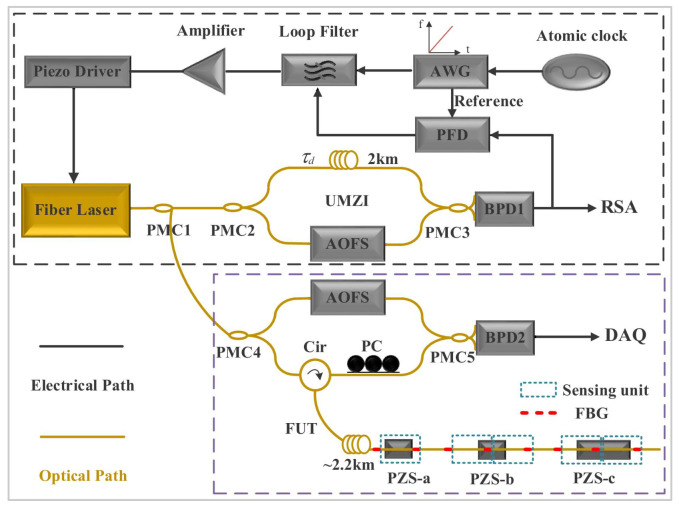
Configuration of the φ-OFDR-based quasi-distributed vibration sensing system, integrating an OPLL-assisted FMCW optical probe with a low-duty-cycle ultra-weak FBG-array sensing fiber for crosstalk-suppressed multi-event vibration detection. Reprinted with permission from Ref. [84] © Optica Publishing Group.

Addressing the limited detection sensitivity and acoustic bandwidth induced by laser phase noise in conventional OFDR, Tan et al. [83] introduced dual Kerr soliton microcombs as the light source, with the schematic shown in Figure 13. This system utilizes frequency multiplexing technology achieving an ultra-high sensitivity of 150 pε/$\sqrt{\mathrm{Hz}}$. This represents a significant breakthrough in dynamic sensing performance.

Quasi-distributed sensing schemes based on weak reflector arrays (WRAs) are widely adopted to mitigate SNR fluctuations caused by Rayleigh fading. Using a φ-OFDR system, Meng et al. [84] employed ultra-weak FBG (UW-FBG) arrays and an OPLL-assisted FMCW optical probe for quasi-distributed vibration sensing, as shown in Figure 13. By matching the grating spacing with the sensing spatial resolution, the system suppressed crosstalk and enabled quantitative multi-event vibration sensing with a spatial resolution of approximately 2.5 cm over about 2.2 km. In practical industrial applications, Qin et al. [85] applied a differential cross multiplication algorithm to a φ-OFDR weak reflector sensing array with square connectors with ultra-physical contact (SC/UPC). This setup successfully monitored power transformer tank vibrations with a 0.112 rad/g sensitivity within 0–1.2 kHz. Xiong et al. [86] proposed a φ-OFDR internal modulation scheme using a distributed feedback laser (DFB) and a 4 cm weak reflector array. This approach achieves a 50 dB SNR at a 4 kHz repetition rate without requiring an AUX. Furthermore, Shi et al. [87] developed a numerical model based on the transfer matrix method (TMM). This model simulates light propagation in OFDR systems with in-line weak reflectors, which provides crucial theoretical validation for signal characteristics. Hu et al. [88] proposed a vibration localization algorithm based on piecewise CC and automatic threshold decision. By comparing the CC curve with an automatic threshold, this method successfully locates piezoelectric transducer (PZT) vibrations on a 27 km fiber.

### 4.3. Fiber Optic Shape Sensing

The core of shape sensing technology based on OFDR lies in utilizing RBS to achieve fully distributed measurements with millimeter or even sub-millimeter spatial resolution. In recent years, research advancements have primarily focused on innovations in special optical fiber structures, parallel measurement methods and algorithm optimization.

Researchers have explored specialized optical fiber designs to enhance the weak and stochastic RBS signals. For φ-OFDR-based shape sensing, Fu et al. [89] employed femtosecond laser inscription to fabricate permanent scatter (PS) arrays within an MCF, boosting the spatial resolution to 200 µm. Nevertheless, focal deviations during inscription can degrade the overall SNR and signal consistency of the system. In a follow-up work [90], the same group extended this strategy by inscribing weak fiber Bragg grating (WFBG) arrays in MCF via femtosecond laser point-by-point writing, which further reduced shape reconstruction errors while simplifying the demodulation process.

**Figure 14:**
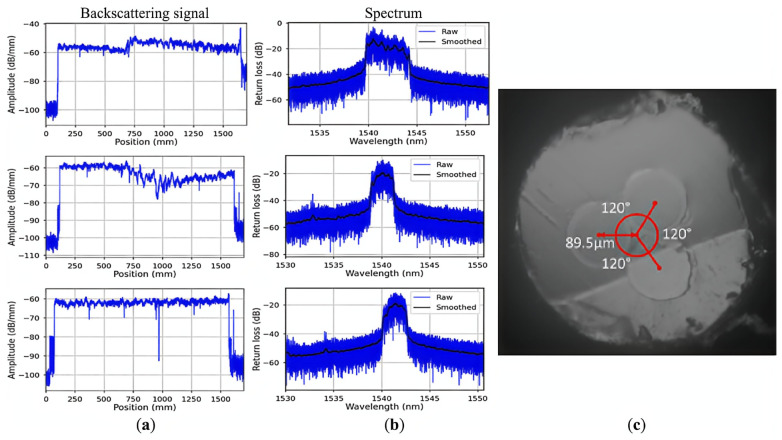
OFDR shape sensing using a polymer-extruded optical fiber triplet with randomly chirped gratings. Adapted with permission from Ref. [91] © Optica Publishing Group. (**a**) Backscattering amplitude traces of the three fibers in the spatial domain; (**b**) corresponding reflection spectra showing the enhanced broadband backscattering responses; and (**c**) microscopic cross-section of the extruded triplet, showing the near-equilateral fiber arrangement with approximately 120° separation and an 89.5 µm core-to-center distance.

As a flexible and low-cost sensing solution, Francoeur et al. [91] proposed an OFDR-based shape-sensing approach for intra-arterial catheter guidance using a polymer-extruded optical fiber triplet with randomly chirped gratings. As shown in Figure 14, the randomly chirped gratings enhance the backscattering response of the three fibers, while the extrusion process forms a flexible triplet with a stable relative core geometry. This structure enables distributed strain measurement and effective 2D/3D shape reconstruction for catheter guidance. The same team later extended this sensor to flexible surgical needle shape sensing for prostate interventions [92]. They optimized the calibration procedure and introduced a tip curvature compensation method, achieving submillimeter-level fully distributed shape reconstruction without prior knowledge of tissue properties or needle deflection. This advancement significantly improves the accuracy and practicality of the sensing scheme in complex soft tissue interventions. Zhang et al. [93] developed a distributed curvature sensing scheme based on helical weak gratings fiber bundles and OFDR. The geometric characteristics of the helical fiber bundle clearly distinguish bending from torsion, and the scheme also optimizes signal demodulation to improve sensing speed.

**Figure 15:**
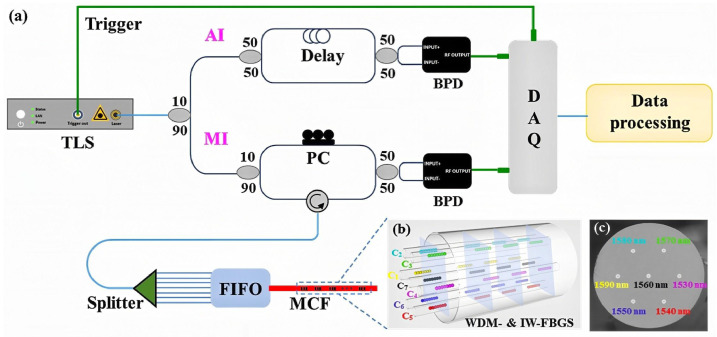
Experimental setup for single-channel OFDR shape sensing based on WDM- and IW-FBG arrays in MCF. Reprinted with permission from Ref. [96] © Optica Publishing Group. (**a**) Optical circuit diagram. (**b**) Longitudinal distribution of gratings along the MCF. (**c**) Central wavelength distribution within the same cross-section of different cores.

Traditional OFDR systems rely on optical switches for sequential core scanning, and the resulting time lag inevitably causes undesirable shape distortion during dynamic measurements. To address this limitation, recent studies have proposed various synchronous multi-core measurement schemes. Liu et al. [94] eliminate this distortion in φ-OFDR shape sensing by designing a frequency-spatial division multiplexing (FSDM) system. This setup uses delay fibers to map core signals to distinct beat frequency bands, enabling simultaneous multi-core measurement with a single laser scan. However, the multi-channel delay fibers increased system complexity. Meng et al. [95] connected two outer cores and the central core in parallel via an optical splitter. This parallel connection enables synchronous acquisition while effectively decoupling temperature and shape crosstalk. Moreover, Meng et al. [96] proposed a single-channel scheme based on wavelength-division-multiplexing (WDM) and identical-weak Bragg grating arrays, as depicted in Figure 15. Each core occupies a different wavelength window, allowing for synchronous strain measurement without optical switches.

In the field of algorithm optimization, research efforts are primarily dedicated to mitigating physical measurement errors such as strain accumulation and multi-core fiber torsion and refining reconstruction accuracy through advanced signal processing. Yin et al. [97] established a coupled strain model for bending and torsion to address the accumulation of strain measurement errors and MCF torsion. They proposed two decoupling algorithms that yielded relative torsion errors of approximately 7% in planar bending. Li et al. [98] established a mathematical model using the Frenet–Serret frame and error propagation theory. This model quantifies the relationship between reconstruction error and key parameters, including curvature, torsion, fiber length and strain measurement error. Subsequently, focusing on φ-OFDR shape sensing using multiple single-core fiber-based sensors (MFS), Li et al. [99] proposed inherent spin rate calibration and external twist compensation methods. These approaches notably improve the reconstruction accuracy by over 20 times and 18 times, respectively. Additionally, they increase the shape reconstruction speed by nearly 10 times relative to the traditional CC method. Bai et al. [100] proposed a first-order differential local filtering algorithm which detects and smooths abrupt points in strain differential signals. It reduced the end error from 1.07–2.97% to 0.20–0.78% for three bending shapes with a low computational cost. Lv et al. [101] employed the iterative closest point (ICP) algorithm to correct coordinate system mismatches between the reconstructed shape and the ground truth, which effectively reduced the 3D shape reconstruction error. But the conventional ICP algorithm relies on initial value estimation and may converge to local optima, and excessive filtering could also obscure minute shape features. In addition, Shan et al. [102] implemented a GPU-accelerated adaptive spectrum method to greatly improve the processing speed, though GPU-based parallel computation may introduce extra hardware costs and power consumption. Lastly, Meng et al. [103,104] proposed the vector projections method, which proved geometrically that only two non-collinear outer cores are sufficient to resolve the curvature vector. They combined this finding with a robust full combination fusion strategy to enhance measurement stability.

### 4.4. Refractive Index and Biochemical Components Sensing

OFDR has been widely used in external refractive index (RI) sensing and the derived biochemical component detection. This subsection first summarizes the optimized fiber microstructures for high-performance OFDR-based RI sensing then introduces its advanced applications in specific biomarker detection and gas/chemical environment monitoring.

**Figure 16:**
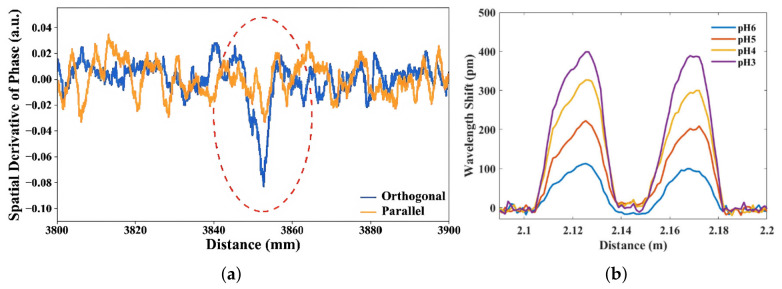
(**a**) Polarization-induced variations in the spatial derivative of the phase across the D-shaped sensing segment. Reprinted from Ref. [110], published by Optica Publishing Group under the Creative Commons Attribution 4.0 License. (**b**) Distributed pH sensing result when the hydrogel-coated fiber is divided into three parts. Reprinted with permission from Ref. [111] © Optica Publishing Group.

Researchers use fiber micro-machining and surface functionalization to interact with external media. Hua et al. [105] used a tapered fiber with a 6 µm taper waist diameter. This setup achieved a high RI sensitivity of 2970 nm/RIU. However, the small waist diameter reduces mechanical strength. Guo et al. [106] solved this balance issue by using a tapered multi-mode fiber (MMF). They excited higher-order modes like the LP_03_ mode increasing sensitivity by 2.4 times compared with the LP_02_ mode while maintaining a 3.5 µm waist. Alternatively, Fu et al. [107] used hydrofluoric acid to etch high-scattering germanium-doped single-mode fiber (Ge-doped SMF) which has a 5 dB higher RBS intensity. They achieved an average sensitivity of 757 GHz/RIU over 10 cm. However, etched fibers often face temperature cross-sensitivity. Zhu et al. [108] proposed an etched MCF scheme for this problem. They used exposed outer cores for RI sensing and an unexposed inner core for temperature compensation. This decoupled measurement reached a maximum sensitivity of 47 nm/RIU. Offering a more robust engineering solution, Xu et al. [109] developed a distributed refractive index sensor using a U-bent SMF. This setup excites higher-order modes and achieves a sensitivity of 39.08 nm/RIU. Bartaula et al. [110] applied φ-OFDR and polarization control to a side-polished D-shaped fiber, achieving 162 nm/RIU sensitivity without the need for post-processing. Figure 16a shows the phase spatial derivative variations.

Surface functionalization extends this technology to biomarker detection. Hua et al. [112] proposed the first distributed optical fiber biosensor based on tapered fiber and OFDR. The authors functionalized the tapered fiber surface with human immunoglobulin G (IgG) through polydopamine (PDA)-assisted immobilization, which enabled the specific recognition and detection of anti-human IgG. As a result, the sensor exhibits a concentration measurement limit of 2 ng/mL for anti-human IgG over an effective sensing range of 50 mm. Guo et al. [113] developed a φ-OFDR-based graphene oxide (GO)-sensitized tapered fiber sensor for carcinoembryonic antigen (CEA). They introduced a differential relative phase demodulation and radius denoising algorithm. This scheme lowered the limit of detection to 1 ng/ml and reached a spatial resolution of 80 µm.

Functional materials swell in response to specific molecules. This volume change converts gas and ion concentrations into measurable strain signals. Liu et al. [114] proposed a multi-point hydrogen sensor which uses OFDR and fiber-tip FP microcavities with a Pd/GO film. The sensitivity is approximately 1 nm/%, and the response time is 12.6 s, which enables long-distance monitoring. Li et al. [115] exploited the swelling of a PI coating on unstripped commercial fibers for CO_2_ sensing. They realized fully distributed and reversible measurements across a 0% to 40% concentration range. Finally, Yin et al. [111] developed a distributed pH sensor coated with a PEGDA-based pH-sensitive hydrogel. The hydrogel stretches the fiber as it swells with decreasing pH. This volume change yields a high sensitivity of −199 pm/pH, as shown in Figure 16b.

### 4.5. Testing of Integrated Photonic Devices

The application of OFDR to optical device characterization was pioneered by Soller et al. [116], who demonstrated a high-resolution, polarization-sensitive OFDR technique for component testing, achieving a 22-µm physical spatial resolution to analyze FBGs and birefringent assemblies. Building upon this foundational paradigm of localized optical metrology, modern device testing has increasingly shifted toward photonic integrated circuits (PICs) on silicon photonics and other platforms. As PICs continuously increase in integration density and functional complexity, testing requirements have evolved from conventional end-to-end transmission-spectrum measurements to spatially resolved characterization of on-chip local structures. In this context, OFDR can retrieve RBS distributions along waveguides from a single-ended access, enabling critical tasks such as propagation-loss evaluation, local defect localization and parameter inversion. Compared with conventional transmission-based methods, OFDR achieves spatial decoupling in complex cascaded circuits with lower alignment dependence.

Zhang et al. [117] first introduced OFDR into the quality-factor (Q) characterization of microring resonators in 2021. They constructed a polarization-diverse OFDR system to obtain the loaded Q factor by attenuation coefficient fitting and solve the intrinsic Q factor using an equation system built by devices with identical structures and different coupling gaps. The measurement results are in good agreement with conventional transmission measurements, verifying the advantages of single-ended access. This method requires structurally consistent devices with varied coupling conditions, which imposes high requirements on process consistency. Tokushima et al. [118] proposed a circuit-level analysis framework combining OFDR with a backscatter model of multiple-reflection paths to address overlapping reflection peaks in silicon photonic circuits. This method establishes a time-equivalent multi-reflection-path model and extracts component transmittance and reflectance by separating key parameters from overlapping reflection peaks. The measured key parameters are highly consistent with 3D finite-difference time-domain simulation results, filling the research gap in OFDR-based transmittance and reflectance measurement for silicon photonic components. The limitation is that the model relies on design consistency assumptions, with reduced applicability in circuits containing heterogeneous components or complex topologies.

**Figure 17:**
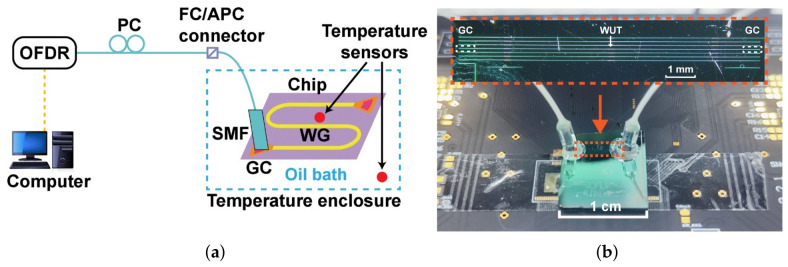
(**a**) Schematic of OFDR-based DTS of SOI chip. (**b**) Image of packaging SOI chips. Embedded image displays the SOI waveguide (WUT) under test. White dashed boxes indicate the grating couplers (GCs). Adapted with permission from Ref. [119] © Optica Publishing Group.

Kong et al. [119] extended the application of OFDR to distributed temperature sensing on SOI chips. They exploited RBS induced by SOI waveguide sidewall roughness and established a mapping between spectral shift and temperature change by combining thermo-optic and thermal expansion effects. The system schematic and packaged SOI chips are shown in Figure 17. This approach achieves 200 µm spatial resolution and 88 pm/K sensitivity in the range of 22.8–200 °C, realizing on-chip temperature-field reconstruction without dedicated sensing structures. However, dispersion-induced spatial resolution degradation with increasing waveguide length restricts its application in large-area on-chip sensing.

With the development of PIC testing from precise characterization of individual devices to statistical assessment of component libraries and high-throughput screening, OFDR has shown a more explicit engineering orientation in 2025 [120,121]. Nawa et al. [120] systematically validated the feasibility of OFDR for characterizing key silicon photonic building blocks including waveguides, bends, crossings and multi-mode interference couplers in their initial work. The method realizes non-destructive single-sample evaluation with approximately 25 µm spatial resolution and greatly reduced testing time consumption. They further optimized this OFDR-based approach to adapt to high-throughput evaluation in subsequent work [121]. They introduced segmented median filtering to mitigate fading noise and established a statistical model based on standard error and 95% confidence intervals to quantify measurement uncertainty. This optimized method further improves the consistency between OFDR-based testing and traditional transmission measurements.

Overall, OFDR-based PIC testing has evolved from single-device parameter extraction to circuit-level parameter inversion and on-chip distributed sensing, with proven engineering value in high-throughput component library evaluation. Key future research directions include alleviating dispersion-induced resolution degradation, realizing robust fading noise suppression and establishing standardized calibration for systematic errors related to coupling and packaging.

### 4.6. Battery Health Monitoring

Batteries exhibit strong spatiotemporal non-uniformity during operation and failure processes. Local temperature surges and stress concentrations usually occur earlier than macroscopic electrical signal anomalies and are closely correlated with dendrite growth, capacity fade and thermal runaway risks. Compared with conventional monitoring relying on a limited number of point sensors, OFDR offers high spatial resolution and continuous deployability along target structures, enabling accurate identification of early abnormal signs including hot spots, local swelling and structural stress concentrations. Existing relevant studies can be broadly divided into two main research directions. The first is distributed temperature monitoring and thermal abnormality early warning for safety and thermal management, and the second is distributed strain and stress monitoring for state estimation and mechanism characterization.

#### 4.6.1. Distributed Temperature Monitoring and Thermal-Abnormality Early Warning

Temperature-field monitoring is central to battery thermal management and early safety warning. OFDR-based distributed thermometry typically realizes spatial sampling along battery cells or modules through dedicated fiber deployment. The key technical challenges in this field include the trade-off between spatial resolution and sampling rate, the suppression of temperature-strain cross-sensitivity and the evaluation of fiber deployment effects on battery heat dissipation and long-term service reliability.

Guo et al. conducted systematic studies on OFDR-based temperature monitoring for lithium batteries and progressively developed the technical system and application scope of OFDR distributed thermometry in a series of works [122,123,124,125]. In 2022, the team proposed a frequency-resolved-multiplex OFDR method to address the challenge of simultaneously achieving high spatial resolution and measurement speed for thermal runaway monitoring [122]. This method divides the laser tuning process into fixed frequency intervals instead of conventional time segmentation and compensates for tuning non-linearity with equal-frequency resampling, realizing 100 Hz distributed temperature measurement with 2 cm spatial resolution and ±0.2 °C accuracy. However, this work only adopted a simulated metal heat source, with no validation performed on actual battery samples. Building on this framework, Guo et al. [123] further realized high-resolution surface thermal imaging of 21700 cylindrical cells in 2023. A bare fiber packaged in an FTE tube is used to isolate strain interference and wound across the full cell surface. The method maps 1D distributed temperature data to 2D thermal images, achieving 0.1 °C temperature resolution. Also, it successfully identifies surface thermal gradients up to about 3.8 °C and periodic temperature patterns correlated with internal electrode structures.

To further increase measurement-point density and quantify circumferential thermal gradients, the team extended this thermal imaging method in 2024 [124]. A PTFE-packaged fiber is spirally wound 63 turns around the surface of a 21700 cell, providing over 1300 sensing points to enable global temperature-field imaging with 3 mm spatial resolution. The maximum temperature difference measured during 1.5 C discharge reaches 8.37 °C, representing about 194% improvement over thermocouple measurements. This work first quantifies the circumferential temperature gradient of cylindrical cells and identifies the positive tab as the main heat accumulation region. In 2025, the same group further expanded this technical system to large-format prismatic cells and proposed millimeter-resolution operando thermal imaging [125]. With a consistent system configuration, the fiber is wound over the full prismatic cell surface, providing over 400 sensing points with 3 mm spatial resolution and ±0.2 °C accuracy. The method achieves a 1025% improvement in temperature difference detection capability over thermocouples during 1.0 C charging and quantitatively reveals the thermal behavior of prismatic cell current tabs and corresponding heat transfer paths.

At the module level, Krause et al. [126] applied OFDR for spatial localization and early warning of thermal abnormalities in a 4p3s battery module. A fiber attached to cell surfaces is used to obtain temperature distributions with 2.6 mm spatial resolution during charge–discharge cycles and customized abuse tests. The system detects local hot spots over 80 °C and identifies thermal abnormalities 30 min earlier than conventional temperature sensors and 15–20 min earlier than electrical signal anomalies. This work is limited to surface temperature measurement, and the impacts of fiber deployment on battery heat dissipation and long-term reliability require further systematic validation.

Different from conventional fiber-optic schemes limited to surface or central-void thermal mapping, Wang et al. [127] proposed an integrated functional electrode (IFE) design that embeds distributed optical fibers into 3D-printed grooved substrates to decouple temperature and strain signals. This electrode–sensor integration achieves high-resolution in situ monitoring across the internal electrode plane without compromising the battery’s electrochemical performance, as evidenced by long-term cycling in both LFP and NCM systems. This work provides a novel, non-damaging pathway for internal battery thermal sensing.

**Figure 18:**
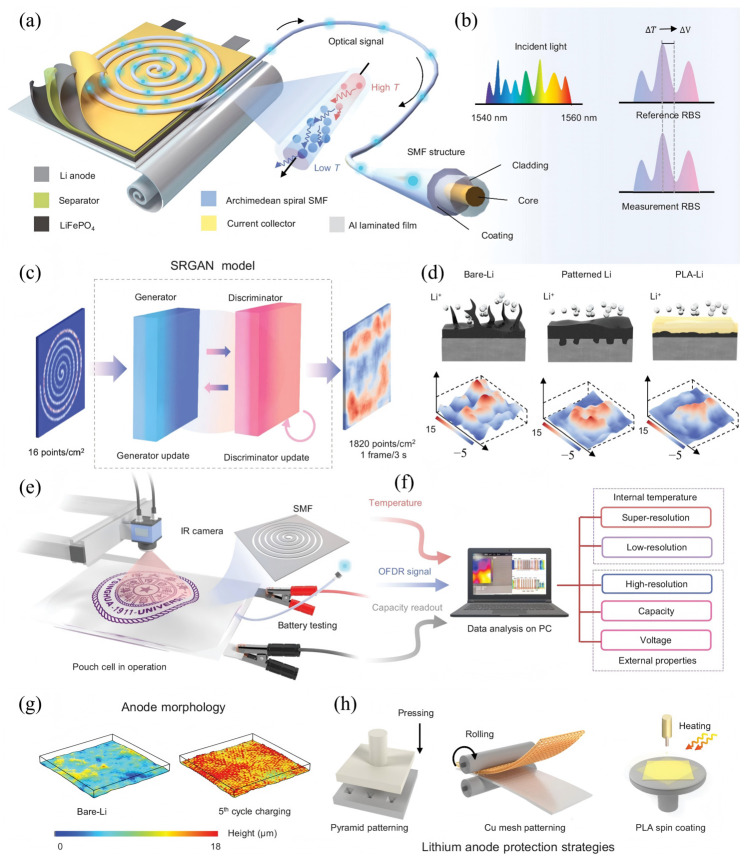
Principle and setup of the OST-SRTM system. Reprinted from [128]. (**a**) LMB pouch cell structure and SMF placement in the cell. (**b**) RBS principle and 1D temperature-position mapping along the fiber. (**c**) 1D-to-2D temperature conversion and super-resolution algorithm for temperature distribution. (**d**) Lithium anode dendrite growth process (upper) and temperature maps after 50 cycles (left to right: Bare-Li, Patterned-Li, PLA-Li). (**e**) Experimental setup: battery test system, IR camera, OFDR system and data processing laptop. (**f**) Correlation and functions of optical, electrical and thermal signal processing. (**g**) Lithium anode morphology simulation. (**h**) Lithium anode protection strategies.

Existing OFDR techniques face challenges including insufficient monitoring dimensionality, limited spatial point density and difficulty in prompt capture of internal thermal events when monitoring lithium-metal batteries. To address these issues, Xun Guan and co-workers proposed an operando spatiotemporal super-resolution thermal monitoring (OST-SRTM) system integrating OFDR distributed thermometry, two-dimensional structured fiber deployment and a super-resolution reconstruction algorithm, with the system schematic shown in Figure 18 [128]. A single-mode fiber fabricated into an Archimedean spiral structure maps 1D continuous sensing points to a 2D planar distribution. A super-resolution generative adversarial network-based algorithm fills inter-fiber sampling gaps, enabling the system to achieve a temporal resolution of 1 frame per 3 s and increase 2D temperature map spatial sampling density from 16 to 1820 points per square centimeter.

The team embedded the spiral fiber behind the copper current collector of a Li/LFP pouch cell, realizing full-lifecycle continuous temperature monitoring without compromising electrochemical performance. The system accurately captures temperature non-uniformity and hot spot evolution during cycling and achieves precise localization and time-resolved visualization of thermal transients in nail-penetration abuse tests. They also quantified the thermal management effects of three lithium anode protection strategies with the proposed system. Compared with conventional destructive diagnostic methods, OST-SRTM exhibits significant advantages of real-time monitoring, non-destructive detection and large-area coverage, with superior comprehensive performance in measurement accuracy and equipment integration. This technique also shows good transferability and provides a new solution for thermal safety monitoring and early warning of lithium-metal batteries.

#### 4.6.2. Distributed Strain Monitoring and Battery State Estimation

Battery volume variation and interfacial stress are key physical observables of internal electrochemical processes, directly correlated with state of charge (SOC) distribution, lithium deposition and active material degradation. OFDR and φ-OFDR deliver high spatial resolution and full phase information utilization, providing a novel pathway for distributed strain field characterization and mechanistic analysis in battery systems.

Li et al. [129] proposed an SOC spatial distribution mapping method by combining OFDR-based distributed strain measurement with deep learning. The method uses OFDR for real-time internal strain monitoring of graphite electrodes and builds a long short-term memory (LSTM) network to map strain, current and voltage signals to SOC, realizing visualization of SOC spatial distribution. Experiments with a fiber-embedded lithium iron phosphate || graphite (LFP || Gr) pouch cell achieve an SOC estimation RMSE of 2.01%, with a 41.2% error reduction compared with models relying solely on electrochemical parameters.

Liu et al. [130] developed a φ-OFDR-based distributed strain-field monitoring scheme for SOC and state of health (SOH) estimation without electrochemical parameters. A complex-domain denoising algorithm is adopted to improve φ-OFDR phase demodulation robustness, and temperature compensation fibers are used to extract distributed strain features while decoupling temperature interference. The team built a feedforward neural network (FNN) for SOC prediction and an LSTM model for SOH prediction, achieving a prediction accuracy of 98.3% for SOC and 96.3% for SOH. However, this method is only validated under fixed temperature and charge–discharge rate conditions, and its robustness under complex operating environments requires further verification. Furthermore, Liu et al. [131] extended φ-OFDR to investigate the chemo-mechanical coupling behavior of SiO/C composite anodes. Using the same sensor deployment as in [130], the team fixes the fiber sensor on the surface of SiO/C composite anodes and embeds it into pouch cells, realizing strain monitoring with 1.5 mm spatial resolution and approximately 1 με accuracy. This work reveals the correlation between electrode stress, charge–discharge processes, SOC and electrode loading, verifies the feasibility of strain-based prediction of key electrode performance and provides experimental evidence for electrode loading optimization and structural design.

Battery health monitoring should also attach great importance to the long-term mechanical and chemical stability of optical fiber sensors. Liquid electrolytes in lithium batteries may degrade or swell standard acrylate fiber coatings during prolonged exposure and cycling, leading to increased optical loss and irreversible baseline drift. Meanwhile, lithium-metal anodes and other high-expansion electrode materials can undergo substantial volumetric variation during cycling, exerting repeated compressive stress on fibers and potentially causing microbending losses or even fiber fracture. Current mitigation strategies mainly include reinforced polyimide coatings, thin metal film encapsulation and flexible polymer jacketing. Although these methods improve sensor durability to a certain extent, they still have inherent trade-offs between packaging robustness, strain transfer fidelity and temperature response speed. Further in-depth research is therefore needed to resolve this key engineering challenge for practical deployment.

## 5. Integration of Artificial Intelligence and Machine Learning in OFDR

In recent years, the convergence of AI and ML with optical sciences has catalyzed a profound paradigm shift across the spectral, imaging and sensing domains. Beyond classical fiber configurations, data-driven approaches have thoroughly revolutionized the broader landscape of intelligent photonics [132], structured and vortex light metrology [133,134] and automated inverse problem solving such as thin-film ellipsometry [135], fundamentally expanding measurement frameworks by unlocking multi-dimensional physical degrees of freedom. Meanwhile, within the established framework of optical metrology, deep learning has demonstrated remarkable success in handling core back-end tasks including signal denoising, phase retrieval and subset correlation [136].

Inspired by these overarching trends, AI and ML have been increasingly introduced into fiber-optic sensing, bringing new opportunities to overcome performance bottlenecks under complex noise and extract high-dimensional sensing information. For OFDR, AI and ML integration mainly focuses on two core directions. The first is to optimize the signal processing chain including denoising, calibration and demodulation to improve sensing resolution, robustness and real-time performance. The other is to extract application-oriented information from distributed sensing data to realize intelligent sensing systems. It should be noted that these two goals are not strictly separated. The performance of low-level demodulation directly determines the data quality for application-level tasks, while the requirements of application scenarios drive the evolution of low-level algorithms.

### 5.1. Demodulation and Signal Enhancement

Current studies on low-level demodulation mainly embed learning models into the conventional OFDR processing chain, forming three representative technical directions. These include direct phase or frequency-shift estimation for improved detection stability, learning-based replacement of computationally intensive modules for faster demodulation and deep network-enabled post-processing denoising for signal enhancement.

Aitkulov et al. [137] proposed a U-Net convolutional network to address signal fading in OFDR-based distributed acoustic sensing. The network directly estimates phase difference from the real and imaginary parts of scattering signals, avoiding phase discontinuities at fading points in conventional homodyne detection. It achieves a 6 dB improvement in phase estimation accuracy and 5.1–7.3 dB SNR improvement for acoustic perturbation detection in experiments. Yin et al. [138] adopted an MLP to replace the conventional cross-correlation algorithm (CCA) for wavelength shift estimation. The MLP learns Rayleigh spectrum fingerprint features to mitigate spectral overlap reduction and multi-peak issues under large strain, formulating strain demodulation as a 17-class discrete classification problem. In a 10 m fiber experiment, this method achieves over 40 times faster processing speed than CCA and maintains 90% classification accuracy for strains exceeding 2600 με. However, its discrete output limits continuous strain estimation, and the preset class set restricts scalability to wider measurement ranges.

Zhang et al. focused on the strain resolution degradation of OFDR under long sensing range and high spatial resolution conditions [139,140,141]. They first proposed a 1D convolutional neural network (1D-CNN) for end-to-end denoising of strain-distance signals [139]. This method avoids complex manual parameter tuning, achieves a strain standard deviation of 1.1 με over a 140 m fiber with 4 mm resolution and improves strain resolution by 6 times. However, insufficient systematic validation leaves its applicability boundary unclear, and the small convolution kernel limits the capture of global noise correlation features in long-range fibers. Building on this prior work, the same group developed a data and physics-driven large kernel denoising network (LKDNet) to break the inherent trade-off between spatial resolution and strain resolution in OFDR [140,141]. Training data of the network are generated based on OFDR sensing physics and a physical noise model, and large-kernel convolution modules are adopted to expand the effective receptive field while retaining local details. This method achieves 0.857 mm spatial resolution and 0.91 με strain resolution over a 140 m range and maintains stable performance under large strains of 1000–2000 με. It provides a clear optimization path in both receptive field expansion and physics-constrained training data construction.

Pedraza et al. [142] proposed a phase-sensitive and polarization analysis OFDR method with a neural network model for temperature and strain decoupling. A deep neural network with densely connected layers is used to regress temperature and strain increments, with explainable AI tools to analyze the network decision logic. This method achieves an absolute medium error of 1.9 K for temperature and 60.1 με for strain in experiments.

Mądry et al. [143] proposed a linear regression-based automated demodulation method for simultaneous temperature and relative humidity (RH) measurement with OFDR. A sensing unit is formed by cascading a bare fiber (temperature-sensitive only) and a PI-coated fiber (responsive to both temperature and RH). A regression model predicts temperature and RH directly from spectral shift and position data, avoiding complex sensitivity-matrix solving with manual calibration. Experiments on a 3 cm sensing unit show a temperature RMSE of 0.36 °C and RH RMSE of 1.73%, with a data processing time of 4 ms. This method outputs the average value of the sensing unit rather than continuous distributed results, limiting its spatial resolution.

Liu et al. [144] introduced an extreme learning machine (ELM) for fast and accurate temperature demodulation, balancing accuracy and processing speed. The single-hidden-layer structure of ELM avoids iterative backpropagation, meeting real-time demodulation requirements. In experiments with a 30–50 °C temperature range and 0.5 °C steps, this method achieves a mean absolute error (MAE) of 0.04 °C, with an average processing time of 0.17 s and training time of 0.94 s.

Huang et al. [145] proposed a distributed temperature demodulation algorithm fusing LSTM and CNN to address limitations of CC and existing ML methods. The training set includes reference spectral segments and spectra generated from a Rayleigh scattering model, reducing the risk of training data invalidation due to system changes. This algorithm outputs the full-position temperature curve in a single pass, with an MAE of only 0.0393 °C and average demodulation time of 0.371 s over a 17.6 m fiber. It partially addresses the data reuse and environmental robustness limitations of lightweight models and realizes a shift from window-by-window to full-position parallel demodulation.

Li et al. [146] proposed a CNN-based image denoising approach to overcome the noise increase caused by reduced CC sliding window size in OFDR systems. The 1D wavelength shift sequence is rearranged into a 2D image for supervised CNN denoising, with peak extraction to reconstruct the strain curve. This method achieves 2 mm spatial resolution and reduces the MAE to 8.2751 με in experiments on a 75 m fiber. Similarly employing 2D rearrangement, Qian et al. [147] proposed a denoising CNN (DCNN)-based enhanced CC strain demodulation method to improve strain accuracy under high spatial resolution and large dynamic strain conditions. The global spectral shift from CC is converted into a 2D image matrix, processed by a 17-layer DCNN to reconstruct accurate strain-gradient distribution. Experiments show good linearity between measured and applied strain in 100–900 με range, with an FUT of 20 m. Its long-range applicability requires further study due to the limited validation distance.

**Figure 19:**
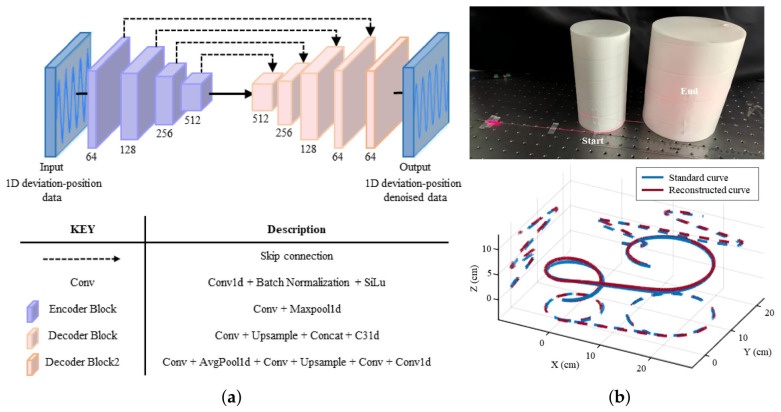
DDSSnet architecture and complex shape reconstruction results. Adapted with permission from Ref. [148] © Optica Publishing Group. (**a**) The U-Net-based DDSSnet for deviation denoising in OFDR shape sensing, featuring encoder–decoder structure and C31d residual connections to eliminate laser phase noise. (**b**) 3D-printed composite mold (two cylinders + straight segment) and its corresponding 3D shape reconstruction via DDSSnet, with a maximum relative error of 1.170%.

In shape sensing, real-time capability and high accuracy are two core requirements. Zhang et al. [148] proposed a fast strain demodulation method based on deviation calculation and deviation denoising for shape-sensing CNN (DDSSnet), as shown in Figure 19a. A U-Net-like encoder–decoder architecture is used for feature extraction and noise filtering, with deviation calculation for initial calibration and residual non-linear tuning phase noise compensation. The 3D reconstruction results are shown in Figure 19b. This method achieves a 9.691 times increase in axial strain-demodulation speed and 9.4 times increase in shape-processing speed, with 2.045 mm spatial resolution and 0.815 με strain resolution.

Overall, low-level demodulation studies show an evolution from localized module replacement to full-chain enhancement. Lightweight and end-to-end models effectively improve processing speed and robustness under extreme conditions [137,138], while deep denoising and data–physics fusion strategies break the resolution trade-off in long-range sensing [140,141]. Multi-parameter decoupling with learning models reduces calibration burden but still lacks systematic validation under wide-range and complex coupling conditions [142,143].

### 5.2. High-Dimensional Inversion and Application-Level Analysis

Application-level tasks focus on extracting high-level semantic information from distributed measurements for scene understanding, structural monitoring and specific engineering applications. These tasks usually have higher-dimensional outputs and are mostly formulated as end-to-end classification or inversion mappings, where the realism and coverage of training data play a critical role in system usability.

Shiloh et al. conducted a series of studies on OFDR-based distributed seismic sensing to address data scarcity and domain shift between simulated and real data [149,150,151]. Their research evolves from generative data augmentation to physics-model-driven data construction. In 2018, they first proposed a generative adversarial network (GAN)-based data augmentation method to adapt simulated data to match real data distribution, achieving 94% binary classification accuracy on a 5 km buried fiber dataset and verifying the feasibility of generative augmentation for seismic event recognition [149]. In 2019, they optimized the framework with a conditional GAN introducing class-conditional constraints to reduce domain shift, extending the task to three-class classification and improving the 5 km scenario accuracy from 80.5% to 83% [150]. But this method still has high false alarm rates due to the insufficient physical fidelity of the generated data. In 2020, they proposed a geophysical-model-driven synthetic data generator, which combines the seismic wave physics model and the OFDR RBS model to produce physically interpretable training data without GAN dependence [151]. This method achieves 92.8% overall accuracy for the three-class classification task using only synthetic training data, greatly reduces false alarms and realizes a critical shift from superficial data imitation to physical mechanism reproduction in training data construction. These studies confirm that constructing physically consistent training datasets matching real data distribution is the core challenge for OFDR-based application-level classification tasks.

**Figure 20:**
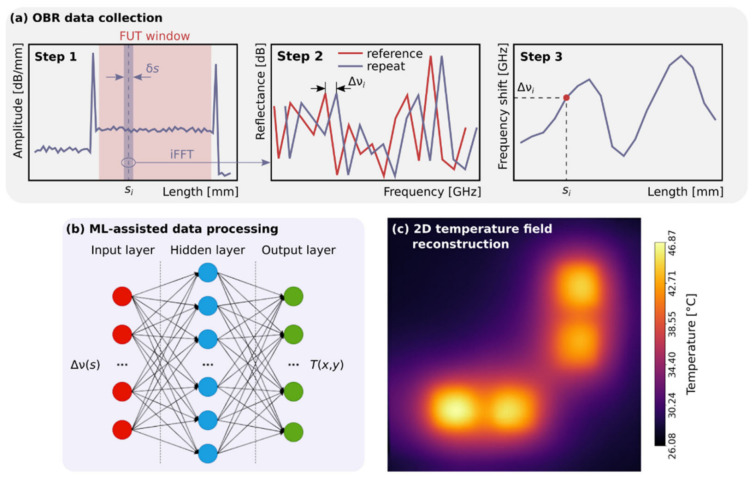
Learning-assisted 2D temperature field reconstruction from OFDR-measured distributed frequency shifts. Reprinted from [152]. (**a**) Distributed frequency-shift extraction along the fiber under test. (**b**) ML-based mapping from spectral-shift sequence to temperature field. (**c**) Reconstructed 2D temperature image.

Wolf et al. [152] proposed a learning-based approach for reconstructing 2D temperature fields from OFDR-measured distributed frequency shifts, as illustrated in Figure 20. In this method, the frequency-shift sequence extracted along the sensing fiber is directly mapped to a 2D temperature image using linear regression or a feed-forward neural network (FFNN), without explicitly requiring the fiber trajectory, panel material properties, or fiber temperature-sensitivity coefficient. Experiments on a 250 mm × 250 mm aluminium panel with a 200 mm × 200 mm fiber sensing area showed that the FFNN achieved an MAE of 0.086 °C and an RMSE of 0.123 °C, demonstrating the potential of learning-based OFDR data processing for 2D field reconstruction.

Silveira et al. [153] developed an OFDR-based smart carpet for plantar pressure analysis. A 17 m single-mode fiber is arranged on a substrate to collect spectral shift signals under different loading states, with a linear mapping between spectral shift and pressure calibrated for plantar pressure distribution calculation. This system achieves 5 mm spatial resolution, with footprint-length estimation relative errors within 2.98% to 5.38% in validation. However, its edge-detection capability for low-pressure signals needs improvement, and the validation population does not include pathological subjects.

Wang et al. [154] proposed a hybrid deep learning model combining a multi-head self-attention temporal convolutional network (MSTCN) and a bidirectional LSTM network (BiLSTM) for 3D shape reconstruction. The model establishes an end-to-end mapping from multi-core fiber strain measurement to spatial coordinates, avoiding error accumulation in traditional Frenet–Serret equation-based methods. Compared with the traditional method, this model improves the optimal reconstruction accuracy by 55.78% and 66.54% for 2D and 3D shape sensing, respectively, with strong environmental robustness.

Overall, application-level studies reflect the trend of OFDR evolving from physical quantity measurement to structural and scene understanding. The core challenges include generalization risks caused by insufficient training data coverage, the requirement for physics-consistency constraints on outputs and system-level real-time requirements. Advances in low-level demodulation and signal enhancement will directly improve the performance of application-level models by increasing input data quality.

The collection of work summarized in Table 8 systematically illustrates the evolutionary trend of AI/ML integration with OFDR technology since 2018. A defining characteristic of this evolution is that AI/ML has evolved from an auxiliary tool for conventional signal processing into a core technique that breaks through the inherent performance bottlenecks of OFDR systems and expands their application boundaries. Research objectives are no longer confined to improving isolated demodulation performance metrics. They are increasingly focused on extracting high-dimensional information such as shape, temperature fields and event categories directly from raw distributed signals. As demands for sensing distance and spatial resolution rise simultaneously, traditional algorithms constrained by fixed receptive fields struggle to capture global noise correlations. This limitation has established large-kernel convolutions and attention structures as key design elements for overcoming the fundamental bottleneck of long-distance high-resolution sensing [140,141]. Meanwhile, physical sensing models are being systematically incorporated into training data generation and network design constraints. This approach enables deep networks to converge stably with limited real measurements, resolving the longstanding concern that data sparsity restricts model complexity [151]. Nevertheless, ensuring the correctness and reliability of training data through rigorous hardware calibration and denoising preprocessing remains a critical prerequisite, as model performance is inherently bounded by data quality. Moreover, purely data-driven networks face inevitable failure risks when encountering out-of-distribution environmental conditions or complex multi-measurand cross-talk.

To mitigate these vulnerabilities and cross-scenario limitations, future research will embed physical priors as network inductive biases to constrain the regression space within reasonable physical limits and improve cross-scenario transferability. Concurrently, adopting uncertainty quantification alongside interpretable structures will enhance decision transparency and provide robust error boundaries for edge cases, while relying on lightweight designs with standardized evaluation benchmarks will balance high accuracy and real-time performance. As these elements gradually mature, AI-integrated OFDR systems will accelerate the transition from laboratory validation to large-scale engineering deployment in areas such as SHM, energy system monitoring and medical rehabilitation.

## 6. Conclusions and Future Prospects

This paper has reviewed the fundamental measurement principles, inherent error mechanisms and core performance metrics of OFDR. Key technological advances over the past five years are systematically surveyed, covering sweep non-linearity calibration, phase noise suppression, spatial resolution enhancement, demodulation algorithm optimization and engineering implementation. Representative applications of OFDR in emerging fields such as battery health monitoring are summarized, and the integration trends of AI and ML methods in OFDR signal processing and application-level information extraction are analyzed. From the traditional performance-improvement perspective, OFDR still faces a systematic trade-off among spatial resolution, sensing range and real-time capability. Wider sweep bandwidth and more accurate resampling help sharpen spatial resolution but substantially increase computational load. Longer sensing ranges generally require higher coherence, stronger noise immunity and more robust polarization-fading suppression. Practical systems must also achieve an appropriate compromise between dynamic range and event detectability. Therefore, future high-performance OFDR will mainly rely on two aspects. The first is the development of TLS with high linearity and low phase noise, as well as more stable coherent detection architectures. The second is the design of high-throughput algorithms and software–hardware collaborative acceleration schemes for large data stream processing. Meanwhile, reliable decoupling of temperature, strain and other physical quantities under multi-physics field coupling remains a key challenge. Achieving this within a single system will require joint design of special optical fibers or functional coatings, polarization diversity detection and model-constrained decoupling algorithms.

The introduction of AI and ML offers new solutions for robust OFDR demodulation under complex noise and multi-scenario adaptive measurement. Existing studies have verified their application potential in signal denoising, physical parameter regression and disturbance event recognition, but their engineering deployment is limited by high data acquisition cost, insufficient cross-scenario generalization and weak model interpretability. Looking forward, a more sustainable route is deep integration of physical mechanisms with data-driven methods. In parallel, building open datasets, unified evaluation metrics and reproducible benchmark tasks will substantially promote the evolution of intelligent OFDR algorithms from isolated demonstrations to comparable, transferable and engineerable solutions.

In addition, integrated OFDR will reshape the technology’s application boundaries and deployment forms. Further integrating key OFDR functional modules onto chips is expected to significantly reduce size, power consumption and cost while improving environmental stability and enabling OFDR to evolve from laboratory instrumentation to field-deployable sensing modules. Key challenges include maintaining on-chip source sweep performance, suppressing packaging-induced system errors and implementing on-chip self-calibration mechanisms. Combined with intelligent adaptive compensation, integrated OFDR will enable low-cost and high-consistency large-scale deployment of the technology.

At the application level, battery health monitoring is the most promising application direction for OFDR. Unlike conventional battery management relying on discrete temperature points and macroscopic electrical signals, OFDR enables direct observation of early battery abnormalities such as hot spots and local structural stress concentration. To promote engineering application, systematic breakthroughs are needed in in situ fiber deployment with reliable packaging, multi-channel system expansion for battery packs and data-mechanism fusion models for battery state estimation and lifetime prediction. With advances in system miniaturization, real-time algorithms and targeted dataset accumulation, OFDR will become a key enabler linking microscopic battery failure mechanisms and macroscopic safety management.

In summary, OFDR is evolving from an ultra-high-resolution optical fiber characterization tool into a comprehensive distributed sensing platform. Driven by the core goals of higher performance, stronger robustness, lower cost and higher intelligence, it will achieve large-scale high-value deployment in key scenarios. Through collaborative innovation in hardware, algorithms and application mechanisms, OFDR will continue to expand its academic frontier and engineering value in DOFS.

## Figures and Tables

**Table 1:** Comparison of representative studies on sweep non-linearity correction.

Ref.	Year	Key Methods/Highlights	Representative Results
[8]	2021	Hilbert transform-based instantaneous frequency extraction, equal-frequency resampling, high-order Taylor correction	12.1 µm physical spatial resolution with 130 nm sweep span and 100 nm/s sweep rate; 21.3 µm physical spatial resolution at the distal end of 191 m FUT (130 nm, 100 nm/s); 1 cm spatial resolution with ±0.15 °C temperature uncertainty over 105 m fiber (75 nm, 100 nm/s)
[9]	2022	High-order Taylor compensation for non-linear sweep noise, Hilbert transform-based random sweep-span drift calibration	±0.51 με/±5.89 με/±19.31 με strain accuracy at 5 mm/1 mm/0.5 mm resolution over 4 m fiber (130 nm, 100 nm/s); ±8.72 με at 1 mm resolution over 104 m fiber (130 nm, 100 nm/s)
[10]	2022	AOM-assisted resampling, fixed frequency shift introduced to increase beat signal zero-crossing rate	Significant physical spatial resolution improvement under short delay conditions verified via multi-method comparison; sweep span: 9 nm; sweep rate: 70 nm/s
[11]	2022	SSWT-based dynamic wavelength calibration, high-precision time–frequency ridge extraction	5 fm calibration accuracy; spatial resolution improved from approx. 100 mm to 1 mm; sensing range up to 80 m; sweep rate expectation: 2.5 GHz/ms; sweep span: 10 nm
[13]	2023	Source-level linearization via high-order sideband injection locking and voltage pre-distortion compensation	60 GHz sweep span with 15 THz/s sweep rate; 5 cm and 7 cm spatial resolution over 1 km and 2 km fiber, respectively; sweep time: 4 ms
[12]	2023	Polynomial regression modeling of non-linear phase noise, matched Fourier transform compensation, improved phase demodulation algorithm	Simulation results: 50 cm spatial resolution over 26.8 km fiber; minimum 0.1 με strain resolution in local segment
[14]	2023	Dual EOFC-assisted frequency synthesis and comb-line stitching for extended sweep range	143 GHz equivalent sweep span; 0.9263 mm physical spatial resolution; sweep rate: 10 GHz/ms
[15,16]	2024	SEFR for simultaneous compensation of sweep nonlinearity and point misregistration, extended to phase-sensitive OFDR	Sensing range extended to 70 m; strain RMSE reduced by up to 41 times under worst LFSR condition; 16 times RMSE reduction for phase-sensitive OFDR; sweep span: 40 nm
[17]	2024	Phase prediction-based adaptive compensation, region-wise virtual reference matching and stitching	0.6 mm physical spatial resolution over 100 m fiber with 20 m delay fiber; sweep span: 36.4 GHz; sweep rate: 70 nm/s

**Table 2:** Comparison of representative studies on phase-noise suppression.

Ref.	Year	Key Methods/Highlights	Representative Results
[18]	2022	PPNE-deskew: periodic phase-noise estimation via moving average filtering and third-order Taylor expansion, and deskew filtering for noise cancellation	535 µm physical spatial resolution over 8 km fiber link; RRP of $1.5\times 10^{7}$, 2.5 times higher than high-order OPLL schemes; sweep span: 3 nm; sweep rate: 5 nm/s
[19]	2023	SSM: segment long sweep; apply PPNE-deskew per segment; spatial position correction and spectral stitching	Sweep span: 20 nm; position error reduced from ~10 m to millimeter level; 1 cm spatial resolution, ±3.2 με strain sensitivity over 1 km fiber; measurable strain range up to 10,000 με; sweep rate: 20 nm/s
[20]	2024	MAI-PNC: multi-arms interferometer for multiple optimum compensation points, segment-wise compensation and distance-domain stitching	2 cm resolution; 3000 με measurable strain range; demodulation RMSE reduced from 0.00310 nm to 0.00035 nm; effective sensing range extended from 104 m to 338 m; sweep span: 10 nm; sweep rate: 100 nm/s
[22]	2025	OPEM coded delay fiber module for on-demand optimal delay; full-range strain measurement via segmented measurement and stitching	Full-range strain distribution acquisition; approximately 24 s per complete measurement
[21]	2025	Residual phase noise reduction via optimized matching between AUX delay fiber and FUT length	2 mm spatial resolution; strain accuracy better than 1.5 με (2σ) over hundred-meter scale fiber; sweep span: 1483–1637 nm

**Table 3:** Comparison of representative studies on signal denoising and enhancement.

Ref.	Year	Key Methods/Highlights	Representative Results
[23]	2021	Polarization control and polarization-diversity reception for SNR enhancement; distance compensation combined with 2D wavelet-threshold denoising of cross-correlation images	2.56 mm spatial resolution over 25 m fiber link; 200 με to 2000 με measurable strain range; ±20 με strain measurement accuracy; sweep span: 1250 GHz; sweep rate: 40 nm/s
[24]	2021	CC imaging processing combined with total variation or 2D Gaussian filtering for noise suppression	1.3 mm spatial resolution over 52 m fiber link; effective resolution of 100–500 με strain steps; sweep span: 1540–1559 nm; sweep rate: 20 nm/s
[25]	2022	BM3D-SAPCA iterative denoising combined with empirical Wiener filtering for long-range sensing	5 cm spatial resolution and 2 με strain resolution over 200 m all grating fiber; significantly reduced measurement error and standard deviation; sweep span: 33.33 nm; sweep rate: 300 nm/s
[26]	2022	PSF extraction from auxiliary interferometer; two-stage Wiener deconvolution for sidelobe-induced ghost peak removal	Cleaner distance trace achieved with low-cost DFB TLS over 26 m test fiber; sweep span: 140 GHz; sweep rate: 175 GHz/s
[27]	2024	Wiener deconvolution in frequency domain, with local Rayleigh-spectrum autocorrelation as PSF	Recovery of noise-buried sensing events at 200 µm ultra-high spatial resolution; sampling time: 0.786 s; sweep span: 1500–1630 nm; sweep rate: 100 nm/s
[28]	2024	AMP method with weighted fusion of multiple morphological opening operations for temperature demodulation	Reliable reconstruction of 43–55 °C temperature gradients over 40 m PI-coated fiber; 3× improvement in gauge length accuracy; 0.563 s processing time; sweep span: 1530–1570 nm; sweep rate: 20 nm/s
[29]	2025	PI-coated fiber combined with adaptive 2D bilateral processing for edge-preserving denoising in humidity sensing	4 mm spatial resolution over 48 m fiber link; 3.1 pm peak-to-peak measurement error, half of conventional processing; sweep span: 20 nm; sweep rate: 10 nm/s

**Table 4:** Comparison of representative studies on demodulation algorithm optimization.

Ref.	Year	Key Methods/Highlights	Representative Results
[30]	2021	Density distribution denoising combined with serial phase correction for robust phase unwrapping in φ-OFDR systems	40 µm physical spatial resolution; 0.1 µm measurement noise; sweep span: 1540–1560 nm; sweep rate: 150 nm/s
[37]	2021	Time-optimized interpolation and distance-domain compensation for position bias correction under large strain	2 mm spatial resolution over 11.2 m fiber; 10,000 με strain range; 76 times faster than conventional processing; sweep span: 1530–1570 nm; sweep rate: 40 nm/s
[31]	2023	Statistical-distribution phase-jump filtering to suppress unwrapping jumps (φ-OFDR)	1.76 με strain accuracy at 20 mm resolution over 30 m FBG array; computation time 3.2% of C-OFDR; sweep span: 1555–1565 nm; sweep rate: 100 nm/s
[32]	2023	Phase prediction for low-SNR segments, phase-jump compensation and interpolation reconstruction for large strain	1 mm spatial resolution; 0–2500 με strain range; 17.32 με measurement error; 0.063 s processing time for 0.8 m fiber; sweep span: 40 nm
[33]	2023	Complex-domain denoising combined with segmented position correction and HNAF backscatter enhancement	0.89 mm spatial resolution; 1.5 με strain RMSE; 2050 με maximum measurable strain; R^2^ of 0.9999; sweep span: 52.8 nm; sweep rate: 100 nm/s
[38]	2023	AZP and DCM under E-SMF	1.5 mm spatial resolution; 9000 με maximum measurable strain; computation time reduced from 138.842 s to 0.476 s; sweep span: 1535–1565 nm; sweep rate: 100 nm/s
[46]	2023	Spectral vernier OFDR with wideband slow scan as main scale and narrowband fast scan as vernier	10 cm spatial resolution; 10,856 με strain range; 1.21 kHz measurement bandwidth; 3σ of 0.4 με; main scale: 1520–1580 nm, 60 nm/s; vernier scale: 35 GHz narrow sweep with 2.42 kHz pulse repetition rate
[34]	2024	Adaptive phase-noise suppression based on adjacent window boundary continuity combined with wavelet packet denoising for φ-OFDR	15.8 µm physical spatial resolution; 1.5 mm sensing resolution; 0.9343 με strain accuracy; 20–270 με effective measurement range; sweep span: 52.4 nm
[35]	2024	FSAV and RVS combined with position correction for coherent fading noise suppression	0.54 mm spatial resolution; 2000 με strain range; 0.87% measurement accuracy; 0.42% RSD in 3 h stability test; sweep span: 2.32 THz; sweep rate: 50 nm/s
[39]	2024	Local spectrum division, quality-factor screening, iterative re-demodulation and weighted fusion for fake-peak error mitigation at strain area edges	1900 με maximum strain over 15 m fiber; RMSE reduced from 0.00878 nm to 0.00026 nm; sweep span: 1545–1555 nm; sweep rate: 100 nm/s
[40]	2024	Self-correcting 2D CC combined with standard deviation dictionary for main peak identification	3 mm spatial resolution at 50 m fiber end; 1000–8000 με strain range; MAE reduced from 14.12 pm to 6.12 pm; sweep span: 1530–1570 nm; sweep rate: 20 nm/s
[41]	2024	Spectral shift adjacent point difference, baseline drift removal, false-peak rejection and interpolation for spurious peak mitigation	7.84 mm spatial resolution over 20 m fiber; 0–10,800 με strain range; ±43.6 με measurement accuracy; sweep span: 1540–1560 nm; sweep rate: 20 nm/s
[44]	2024	MHS time–frequency analysis replacing STFT for improved time–frequency concentration	Spatial resolution improved from 5.0 mm to 0.1 mm; 0.515 sensing quality factor; linear response up to 1500 με
[36]	2024	BEOF combined with ALFEM for simultaneous strain range extension and demodulation speed improvement	400 µm spatial resolution; 4800 με strain range; 1.7 με strain resolution; demodulation time reduced to 25% of conventional method; sweep span: 1540–1550 nm; sweep rate: 20 nm/s
[42]	2025	SN-CC based on local FSR for spurious peak suppression caused by unequal amplitude weighting	3.2 mm spatial resolution at 3000 με strain (sweep span: 10.5 nm); 5.6 times resolution improvement over conventional method
[43]	2025	Modified LCS algorithm mapping 1D spectrum to 2D similarity image for demodulation	18.7 times strain range improvement over NCC; $R^{2}$ of 0.9997; sweep span: 2 GHz; TLS sweep period: 1 ms
[45]	2025	Modified-kernel Cohen’s class time–frequency analysis for ultra-high spatial resolution	Spatial resolution improved from 6.05 mm to 0.16 mm over 14 m fiber; $2.91\times 10^{-3}$ nm RMSE over 0–1000 με range; sweep span: 1545–1555 nm; sweep rate: 100 nm/s

**Table 5:** Comparison of representative studies on computational efficiency improvement.

Ref.	Year	Key Methods/Highlights	Representative Results
[47]	2021	GPU-parallel demodulation kernels and double-buffered pipeline	Approximately 81 times speedup compared with CPU; 60 Hz real-time dynamic strain sensing over 200 m fiber at 20 cm spatial resolution; measurement frame period: 141 ms; sub-frame interval: 16.6 ms; sweep span: 49.8 nm; sweep rate: 500 nm/s
[50]	2023	WDDA for rapid localization, combined with LCC for targeted demodulation	5.3–6.4 times demodulation speedup; effective detection of strain larger than 10 με; sweep span: 1545–1555 nm; sweep rate: 100 nm/s
[51]	2023	DDA based on differential spectrum variance thresholding, with local cross-correlation only for selected strained segments	Approximately 7.18 times demodulation speedup (4575.7 ms → 637.1 ms) over 40 m fiber at 8 mm resolution; minimum detectable strain of 7.81 με; sweep span: 1545–1555 nm; sweep rate: 100 nm/s
[48]	2024	Full FPGA-integrated scheme	Processing time reduced from 1436 ms to 67 ms compared with CPU; 3.49 με strain standard deviation at approximately 0.28 mm resolution
[49]	2024	GPU acceleration for multi-core-fiber (MCF) shape reconstruction	Nearly 21 times reduction in 2D/3D shape reconstruction time; maximum reconstruction errors of 3.23% (2D) and 2.47% (3D) at 5 mm resolution; sweep span: 1525–1575 nm; sweep rate: 150 nm/s
[52,53]	2025	Enhanced BFE for direct peak offset estimation, eliminating dense interpolation operations in peak detection	More than 17 times improvement in demodulation efficiency; sweep span: 40 nm

**Table 6:** Comparison of representative studies on system hardware optimization.

Ref.	Year	Key Methods/Highlights	Representative Results
[54]	2022	Femtosecond-laser inscription for RBS enhancement of more than 40 dB	4.8 cm spatial resolution; strain RMSE less than 2.70 με with low-cost 1 nm TLS; sweep rate: 100.3 nm/s
[55]	2023	UV-exposed E-SMF for intrinsic SNR improvement	37.3 dB scattering enhancement; 2.0 mm spatial resolution; effective demodulation of 200–2600 με strain; sweep span: 10 nm; sweep rate: 80 nm/s
[56]	2023	Integrated with WRFBG array, direct peak searching instead of CC	38 Hz response speed; effective monitoring of strain steps exceeding 25,000 με; accurate rebar yield point localization; sweep span: 1540–1580 nm
[57]	2023	Single-interferometer SCM: arc end of FUT replaces AUX	3 mm/5 mm spatial resolution over 108 m/170 m fiber; 1.34 GHz/°C temperature sensitivity; sweep span: 10 nm; sweep rate: 80 nm/s
[58]	2024	Combine AUX and gas absorption cell in one channel; decouple via filtering/EMD	Reduced hardware complexity and cost; precision 0.106 mm/0.119 mm via filtering/EMD on a 50 m fiber; sweep span: 1530–1570 nm; sweep rate: 200 nm/s
[60]	2024	Fully integrated OFDR system on SOI photonic platform; on-chip interferometer integration, MMI coupler and heterodyne detection	8.28 µm physical spatial resolution with 43 nm sweep range, close to theoretical limit; chip footprint of approximately 1.1 mm × 0.5 mm; sweep rate: 100 nm/s
[59]	2025	Compressive sensing scheme with coprime non-uniform sampling via dual unbalanced AUXs; sparse signal reconstruction	200 m sensing distance with 1 mm spatial resolution; zero-crossing points reduced by an order of magnitude; sweep span: 1539.75–1559.75 nm; sweep rate: 20 nm/s

**Table 7:** Comparison of representative studies on special-effect compensation.

Ref.	Year	Key Methods/Highlights	Representative Results
[61]	2023	Distributed PMF birefringence demodulation based on Rayleigh spectrum shift difference between fast and slow axes	5 cm spatial resolution over 2257 m PMF; $6.8\times 10^{-7}$ measurement uncertainty; effective identification of fiber winding defects
[62]	2024	Dynamic birefringence delay correction combining coarse pre-correction and point-by-point fine compensation for S/P axis alignment	10 cm spatial resolution over 5020 m PMF; $1.4\times 10^{-7}$ measurement accuracy; 1 s processing time per sweep; sweep span: 1545–1555 nm; sweep rate: 10 nm/s
[63]	2024	Distributed chromatic dispersion compensation based on mismatch factor, with quadratic phase fitting and segment-wise compensation	Physical spatial resolution improved from 5.9 mm to 40.7 µm over 500 m RC SMF (20 nm sweep span); resolution better than 15 µm for integrated chip reflection analysis (160 nm sweep span)
[64]	2024	Hybrid polarization-diversity scheme with 45° reference path polarizer and PMF, with peak search and frequency-shift correction	Polarization fluctuation reduced from 40.93° to 2.81° over 1480–1640 nm sweep span; 6 µm physical spatial resolution; −145 dB sensitivity; sweep rate: 40 nm/s

**Table 8:** Summary of AI/ML integrated OFDR systems.

Ref.	Year	Key Methods/Highlights	Representative Results
[149]	2018	GAN data augmentation with physical model synthesis, training improved VGG16 (FiberNet) for binary classification	94% accuracy for footsteps vs. noise on 5 km buried fiber, outperforming simulation-only (50%) or limited real data (70%); TLS sweep frequency: 2 kHz
[150]	2019	Optimized SimGAN architecture with fiber segment parallel processing, extended to 3-class classification	94% accuracy for footsteps, 100% for vehicles at 5 km (TLS sweep frequency: 2 kHz); overall accuracy improved from 42% to 80.2% at 20 km (TLS sweep frequency: 1 kHz)
[151]	2020	Geophysics-driven synthetic data generation integrating seismic and OFDR models, GAN-free	92.8% 3-class accuracy, outperforming simplified simulation (68.6%) without extensive field calibration; scanning repetition rate: 768 Hz
[137]	2022	U-Net CNN directly estimating phase difference from scattering signal I/Q components to mitigate fading	6 dB phase estimation accuracy improvement, 5.1–7.3 dB SNR enhancement for acoustic detection
[152]	2022	Linear regression and FFNN mapping 1D wavelength shifts to 2D temperature fields with bicubic interpolation	FFNN achieved MAE = 0.086 °C, RMSE = 0.123 °C over 20–70 °C temperature range; sweep span: 10 nm; sweep rate: 100 nm/s
[138]	2023	MLP replacing cross-correlation algorithm, formulating strain demodulation as 17-class classification	>40× faster than CCA over 60–2900 με range, maintaining 90% accuracy above 2600 με; sweep span: 5.04 nm; sweep rate: 40 nm/s.
[139]	2023	20-layer 1D-CNN for end-to-end denoising of 1D strain-distance signals	1.1 με strain standard deviation over 140 m fiber with 4 mm resolution, 6× resolution improvement; sweep span: 20 nm
[142]	2023	φ-PA-OFDR dual polarization acquisition, dense neural network for temperature-strain decoupling with XAI analysis	1.9 K absolute medium error for temperature, 60.1 με for strain on single-mode fiber
[144]	2023	Extreme learning machine (ELM) for fast temperature demodulation without iterative backpropagation	MAE = 0.04 °C for 0.5 °C intervals, 78.6% reduction vs. CCA; 0.17 s processing time; sweep span: 16.384 nm; sweep rate: 100 nm/s
[146]	2023	Wavelength shift to 2D image conversion, CNN image denoising for spatial resolution improvement	2 mm resolution over 75 m fiber, strain MAE reduced to 8.2751 με, outperforming Gaussian filtering; sweep span: 1530–1570 nm; sweep rate: 10 nm/s
[140,141]	2024	Data and physics-driven large kernel denoising network (LKDNet) with expanded receptive field, breaking the spatial-strain resolution trade-off	0.857 mm spatial resolution and 0.91 με strain resolution over 140 m; strain resolution improved from 24.16 με to 0.91 με (26×); sweep span: 140 nm
[143]	2024	Cascaded bare and PI-coated fibers, linear regression for automatic temperature–RH decoupling	3 cm sensor achieved 0.36 °C RMSE for temperature, 1.73% RMSE for RH; 4 ms processing time; sweep span: 1545.445–1588.034 nm
[145]	2024	LSTM-CNN fusion network with LSTM for localization/temporal features, CNN for temperature demodulation	MAE = 0.0371 °C per position, 0.371 s full-range demodulation time, 1.37–38.19× speedup; sweep span: 16.384 nm; sweep rate: 100 nm/s
[147]	2025	17-layer DCNN denoising 2D global spectral shift images for high dynamic strain accuracy	98.27% demodulation accuracy at 300 με, 16 mm resolution; R^2^ = 0.99 over 100–900 με range; sweep span: 1530–1570 nm; sweep rate: 40 nm/s
[148]	2025	DDSSnet (U-Net + C31d residuals) for fast strain demodulation from deviation-position data	9.691× faster strain demodulation, 9.4× faster shape processing; 0.581% max error for cylinder reconstruction
[153]	2025	OFDR smart carpet with DBSCAN footprint extraction and linear regression pressure mapping	5 mm spatial resolution, 2.98–5.38% footprint length error, accurate plantar pressure measurement; sweep span: 1525–1610.17 nm
[154]	2025	MSTCN-BiLSTM hybrid network for end-to-end shape reconstruction from multi-core fiber strain	55.78% improvement in 2D accuracy, 66.54% in 3D; strong robustness across SNR conditions

## Data Availability

No new data were created or analyzed in this study. Data sharing is not applicable to this article.

## Abbreviations

The following abbreviations are used in this manuscript:

AC	Alternating Current
AI	Artificial Intelligence
ALFEM	Adaptive Local Feature Extraction and Matching
AMP	Adaptive Morphological Processing
AOM	Acousto-Optic Modulator
AUX	Auxiliary Interferometer
AZP	Adaptive Zero Padding
BEOF	Backscattering Enhanced Optical Fiber
BFE	Buneman Frequency Estimation
BiLSTM	Bidirectional Long Short-Term Memory
BM3D-SAPCA	Shape-Adaptive Principal Component Analysis Block-Matching Three-Dimensional Filter
CC	Cross-Correlation
CCA	Cross-Correlation Algorithm
CEA	Carcinoembryonic Antigen
CMOS	Complementary Metal-Oxide Semiconductor
CNN	Convolutional Neural Network
DAQ	Data Acquisition
DAS	Distributed Acoustic Sensing
DC	Direct Current
DCM	Distance Compensation Method
DCNN	Denoising Convolutional Neural Network
DDA	Dispersion Degree Analysis
DDSSnet	Deviation Denoising for Shape-Sensing Convolutional Neural Network
DFB	Distributed Feedback
DOFS	Distributed Optical Fiber Sensing
E-SMF	Exposed Single Mode Fiber
ELM	Extreme Learning Machine
EMD	Empirical Mode Decomposition
EOFC	Electro-Optic Frequency Comb
FBG	Fiber Bragg Grating
FFNN	Feed-Forward Neural Network
FFT	Fast Fourier Transform
FMCW	Frequency-Modulated Continuous-Wave
FOG	Fiber Optic Gyroscopes
FP	Fabry–Perot
FNN	Feedforward Neural Network
FPGA	Field-Programmable Gate Array
FSAV	Frequency-Shift Averaging
FSDM	Frequency-Spatial Division Multiplexing
FUT	Fiber Under Test
GAN	Generative Adversarial Network
GC	Grating Coupler
Ge-Doped SMF	Germanium-Doped Single-Mode Fiber
GO	Graphene Oxide
GPU	Graphics Processing Unit
HNAF	High Numerical Aperture Fiber
ICP	Iterative Closest Point
iFEM	Inverse Finite Element Method
IgG	Immunoglobulin G
IFFT	Inverse Fast Fourier Transform
IMF	Intrinsic Mode Function
LCC	Local Cross-Correlation
LCS	Longest Common Substring
LFP||Gr	Lithium Iron Phosphate||Graphite
LFSR	Laser Frequency Sweep Range
LKDNet	Large Kernel Denoising Network
LO	Local Oscillator
LSTM	Long Short-Term Memory
MAE	Mean Absolute Error
MAI-PNC	Multi-Arms Interferometer Phase Noise Compensation
MCF	Multi-Core Fiber
MFS	Multiple Single-Core Fiber Bundles
MHS	Margenau Hill Spectrogram
ML	Machine Learning
MMF	Multi-Mode Fiber
MMI	Multi-Mode Interference
MSTCN	Multi-Head Self-Attention Temporal Convolutional Network
NCC	Normalized Cross-Correlation
OFDR	Optical Frequency-Domain Reflectometry
OPEM	Coded Delay Fiber Module
OPLL	Optical Phase-Locked Loop
OST-SRTM	Operando Spatiotemporal Super-Resolution Thermal Monitoring
OTDR	Optical Time-Domain Reflectometry
PD	Photodetector
PDA	Polydopamine
PEGDA	Polyethylene Glycol Diacrylate
PELT	Pruned Exact Linear Time
PI	Polyimide
PIC	Photonic Integrated Circuit
PMF	Polarization-Maintaining Fiber
PPNE-deskew	Periodic-Phase-Noise-Estimated deskew
PSF	Point Spread Function
PZT	Piezoelectric Transducer
RBS	Rayleigh Backscattering
RC-SMF	Reduced-Cladding Single-Mode Fiber
RH	Relative Humidity
RI	Refractive Index
RIA	Radiation-Induced Attenuation
RMSE	Root Mean Square Error
RRP	Range-Resolution^−1^ Product
RVS	Rotated-Vector Sum
SC/UPC	Square Connectors with Ultra-Physical Contact
SCM	Self-Compensation Method
SEFR	Synchronous Equal-Frequency Resampling
SHM	Structural Health Monitoring
SMF	Single-Mode Fiber
SN-CC	Segmented Normalized Cross Correlation
SNR	Signal-to-Noise Ratio
SOC	State Of Charge
SOH	State Of Health
SOI	Silicon-On-Insulator
SSM	Spectral Splicing Method
SSWT	Synchrosqueezed Wavelet Transform
STFT	Short-Time Fourier Transform
TLS	Tunable Laser Source
TMM	Transfer Matrix Method
TSSOF	Tight-Sheath Optical Fibers
UW-FBG	Ultra-Weak Fiber Bragg Grating
WDM	Wavelength Division Multiplexing
WDDA	Wavelength Domain Differential Accumulation
WRA	Weak Reflector Arrays
WRFBG	Weak Reflection Fiber Bragg Grating Sensor
φ-OFDR	Phase-Sensitive Optical Frequency-Domain Reflectometry
